# Design of natural frequency solution method based on the lateral disturbance modeling of slurry discharge pipelines in slurry shield machines

**DOI:** 10.1038/s41598-026-49111-z

**Published:** 2026-04-16

**Authors:** Xingchun Li, Ziliang Li, Yongjia Chen, Yu Cheng

**Affiliations:** School of Electronics and Information Engineering, Wuyi University, Jiangmen, Guangdong People’s Republic of China

**Keywords:** Deflection modeling, Natural frequency solution, Fluid-structure interaction, Dynamics of slurry discharge pipeline, Energy science and technology, Engineering

## Abstract

This study applies classical fluid-structure interaction (FSI) principles to analyze the lateral deflection and natural frequency of slurry discharge pipelines in slurry shield machines. By idealizing the pipeline as an infinitely long cylinder supported by equally spaced rigid rings and adopting the Euler-Bernoulli beam theory and classical fluid-structure interaction principles, the control differential equation considering the interaction between the pipeline and the flowing slurry was adapted for the tunneling environment. This model incorporates the dynamic coupling between symmetrical and asymmetrical vibration modes driven by slurry flow velocity and Coriolis force. Through analytical solutions and numerical methods, the natural frequencies and mode shapes were determined. The results show that the slurry flow will reduce the natural frequencies and may lead to system instability at high flow rates. The accuracy of the model was verified by using the measured data from the site of the South-to-North Water Diversion Project in Beijing, China, spanning four different geological strata. The relative error ranged from 1.58 to 6.29%. The results highlight the dominance of low-order modes and demonstrate that the true engineering value lies in predicting how geological conditions and slurry impurities significantly influence the pipeline’s dynamic behavior, providing guiding suggestions for the engineering design and vibration control of similar systems.

## Introduction

Slurry shield tunneling is a widely used tunnel construction method in soft soil conditions. Its core lies in balancing the pressure of soil and water through pressurized slurry to maintain the stability of the excavation face^1–3^. Slurry is usually a mixture of bentonite and water, circulated through the excavation face and transported to the surface via the slurry discharge pipeline^4^. The slurry discharge pipeline plays a crucial role in removing excavated waste soil, maintaining the performance of the slurry and ensuring construction efficiency^5^. However, during operation, the slurry discharge pipeline is affected by various dynamic loads, including pressure fluctuations of the slurry pump, fluid-structure interactions caused by slurry flow, and changes in geological conditions. These factors may trigger significant vibrations^6–8^. For instance, studies have shown that the presence of large particle debris in gravel formations can lead to a pipeline wear rate ranging from 2.1 mm/100 rings to 3.6 mm/100 rings, thereby exacerbating the risk of vibration-induced fatigue^9–13^. Excessive vibration in pipeline systems can pose significant threats to their long-term integrity and functionality. One of the primary concerns is structural fatigue, which occurs when materials undergo repeated cycles of stress and strain due to continuous vibration. Over time, this can weaken the pipeline’s structure, potentially leading to cracks or complete ruptures. Additionally, operational efficiency can be significantly reduced in the presence of excessive vibration. Vibration can cause increased friction and energy loss within the system, leading to higher energy consumption and reduced flow rates. This not only impacts the overall performance of the pipeline, but may also result in increased maintenance costs and downtime. In severe cases, unchecked vibration can ultimately lead to the failure of the pipeline system. This can have catastrophic consequences, including the release of hazardous materials, environmental damage, and costly repairs or replacements^14,15^. Therefore, a thorough understanding of the dynamic behavior of the slurry discharge pipeline, especially its lateral disturbance and natural frequency, is crucial for ensuring the safety and reliability of the tunneling process.

Although the significance of this issue has been widely recognized, existing research still has deficiencies in accurately capturing the complex interaction between pipelines and slurry flow^16–18^. Many studies have overlooked key factors by simplifying the problem, such as the Coriolis force, the coupling between different vibration modes, or the dynamic effects of particles in the slurry^6^. For instance, early models often assume that the slurry is a homogeneous fluid and fail to fully consider the contribution of particle settlement and wall sliding in the gravel or clay layers to pipeline vibration^19,20^. Recent studies have begun to adopt the coupled method of Computational Fluid Dynamics (CFD) and Discrete Element Method (DEM) to simulate the fluid-particle interaction in the slurry discharge pipeline^21–24^. However, these models mainly focus on pressure loss or wear prediction, lacking a comprehensive analysis of the lateral disturbance and natural frequency of the pipeline^25^. In addition, the influence of geological conditions on slurry infiltration and pipeline dynamic response has not been systematically analyzed either^26^. Unlike previous studies that primarily focused on wear prediction using CFD-DEM coupling, this paper focuses on the analytical solution of lateral deflection and vibration stability. Specifically, by incorporating Coriolis forces and curvature effects, we uncover the coupling mechanism between symmetric and asymmetric modes, which has been rarely explored in existing simplified models. This study aims to fill these research gaps by developing a detailed mathematical model that incorporates Coriolis forces, mode coupling, and the influence of geological conditions.

Research on slurry pipeline dynamics has progressed significantly with the development of sophisticated numerical methods. Recent studies have adopted CFD-DEM (Computational Fluid Dynamics-Discrete Element Method) and even CFD-DEM-FEM coupled simulations to explicitly model the complex fluid-particle-structure interactions. For instance, Fang et al. (2025)^9^ and Yang et al. (2023)^24^ successfully utilized these high-fidelity approaches to assess vibration and wear characteristics, capturing micro-scale details of particle impact and local wall deformation.

However, while these state-of-the-art numerical methods are powerful, they are inherently computationally expensive and time-consuming, making them less suitable for broad parametric studies and rapid design-stage scoping. In the preliminary design phase of tunneling projects, engineers require efficient tools to quickly estimate natural frequencies and identify stability boundaries under a wide range of flow conditions and geological parameters. This represents the distinct niche for the analytical beam-model approach proposed in this study. Unlike complex simulations where the underlying physical mechanisms can sometimes be obscured by massive datasets, the analytical solution provides fundamental insights into the system’s dynamics. It allows for the mathematical decoupling of specific instability drivers — such as the Coriolis force and centrifugal effects — offering a complementary methodology that guides vibration control strategies before detailed numerical verifications are performed. Crucially, it should be noted that while the inclusion of the Coriolis force follows classical FSI theory for pipes conveying fluid (Païdoussis, 1998)^27^, the distinct novelty of this work lies in its application and investigation within the specific, complex context of a solid-liquid slurry flow in a tunneling environment.

To achieve this goal, we model the slurry discharge pipeline as an infinitely long cylinder supported by equally spaced rigid rings, and describe its bending behavior using the Euler-Bernoulli beam theory^28–30^. By applying the energy method and the Hamiltonian variational principle, the control differential equation considering the kinetic energy and strain energy of the pipeline and the slurry was derived, and dynamic effects such as slurry flow velocity, vertical acceleration and Coriolis force were incorporated^31,32^. Subsequently, the model was solved through analytical and numerical methods to obtain the natural frequency and mode shape, with particular attention paid to the influence of the slurry flow rate and the coupling of symmetric and asymmetric modes^8,33–35^. This study also analyzed the influence of different geological conditions (such as gravel, hard rock, and clay layers) and impurities in the slurry (such as slurry clumps and large particles) on the vibration characteristics, revealing the potential role of filter cake formation and particle concentration on the dynamic behavior of the pipeline^36,37^. In summary, rather than proposing a fundamentally new theory, this paper presents a pragmatic analytical framework adapting the classic FSI theory for calculating the natural vibration frequencies of slurry discharge pipelines. Unlike existing engineering practices in tunneling that often simplify fluid-structure interactions, our approach applies the well-established concepts of Coriolis forces and the dynamic coupling between symmetric and asymmetric vibration modes to the specific context of slurry shield tunneling. Furthermore, the primary contribution of this proposed framework lies in providing a more accurate representation of pipeline stability under varying geological strata and flow conditions, validated by actual field data. This offers a practical theoretical tool for vibration control in slurry shield tunneling projects. To verify the validity of the model, we applied it to the site of the South-to-North Water Diversion Project in Beijing and compared the calculated natural frequencies with the experimentally measured values. The results show that the relative error of the model prediction ranges from 1.58% to 6.29%, confirming its reliability in predicting the dynamic behavior of pipelines under different operating conditions and indicating that the model can adapt to complex construction environments.

## Problem description and modeling of deflection dynamics equations

**Fig. 1:**
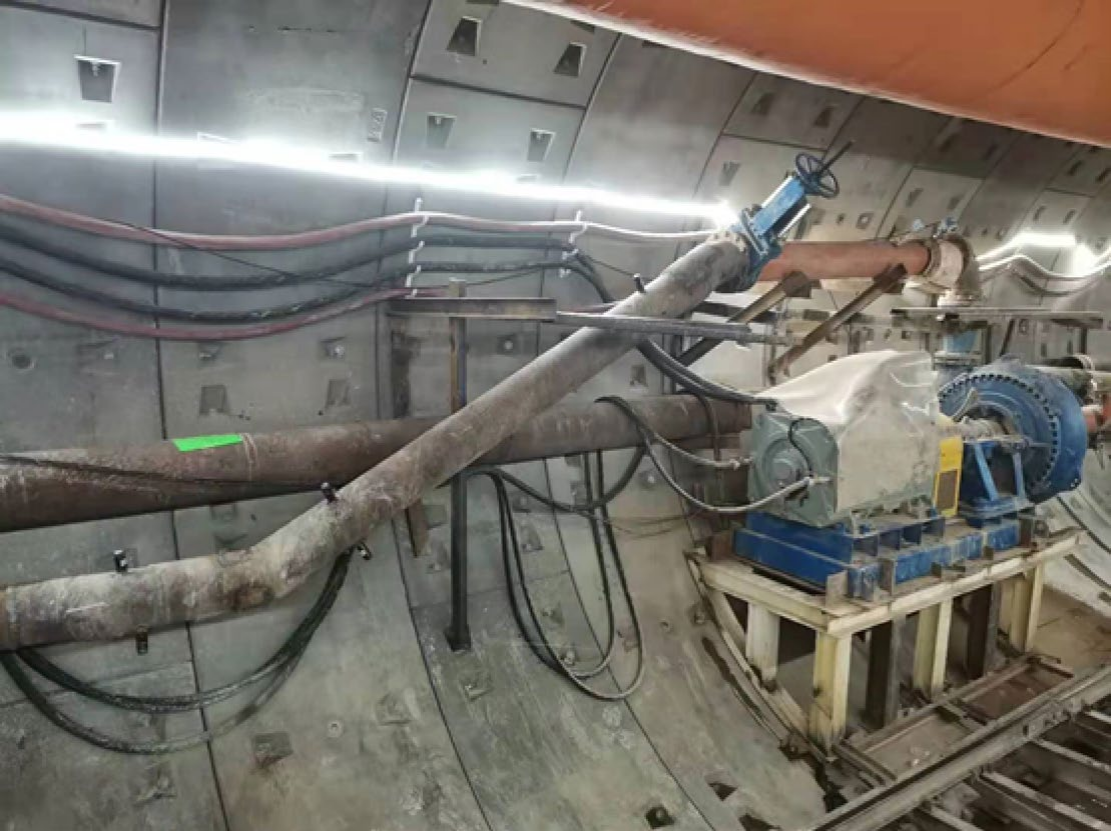
The slurry discharge pipeline for the slurry shield machine.

The physical object of the slurry discharge pipeline for the slurry shield machine is shown in Fig. 1. Ideally, the actual slurry conveying pipeline is an infinitely long cylinder, and it is supported by circular rings at equal intervals. Assuming that the stiffness of the supporting circular rings is large and the width is small, the radial deflection of the pipe wall at the support position is zero, and the differential of the radial deflection along the axial direction of the pipeline is generally not equal to zero.

**Fig. 2:**
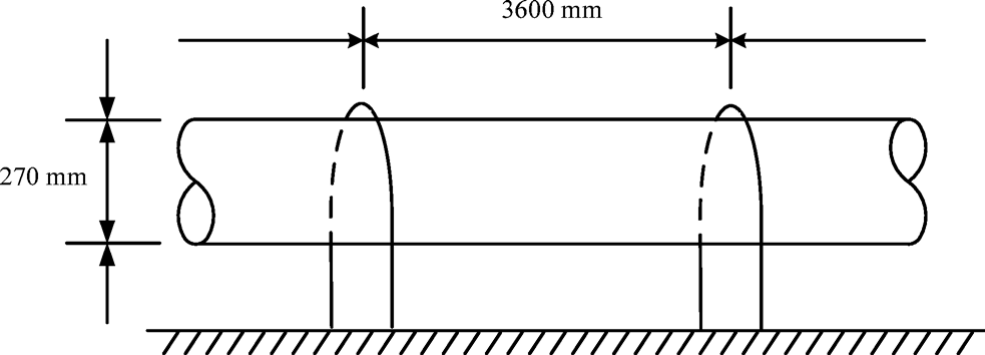
Geometric dimensions of the single-span pipeline.

The geometric parameters of the single-span pipeline are shown in Fig. 2. When the slurry discharge pipeline of the slurry shield is operating under full pipe load, the flow rate of the slurry in the pipe is v, mass per unit length is m (the sum of the mass of the pipeline per unit length and the mass of the slurry inside the pipeline per unit length), the mass of slurry per unit length of pipe is *ρ*, the moment of inertia of the pipe section is*I*, the elastic modulus of the material is *E*.

**Fig. 3:**
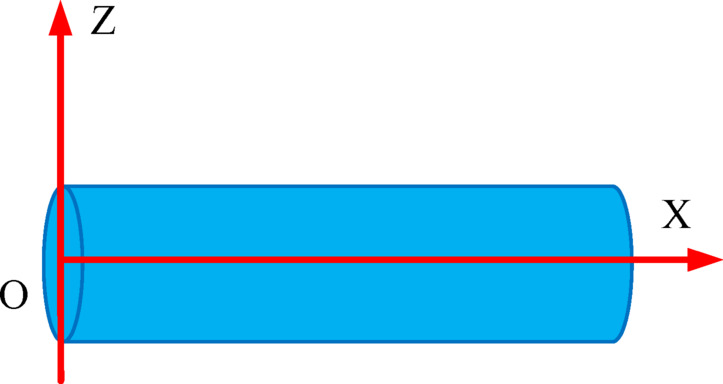
System reference coordinates.

Take the direction of the axis of the undeformed slurry discharge pipeline as the x-axis (positive to the right), and take the direction perpendicular to the x-axis within the symmetry plane as the z-axis (positive upwards), as shown in Fig. 3. For continuous elastic bodies like pipes, which are Euler-Bernoulli beams with constant cross-sections, the strain energy is related to the displacement at all points along the pipe. Since there are an infinite number of points, the displacement forms a curve and must be represented by a function. When a pipeline vibrates transversely, its deflection varies with time and can be represented as $\:z\left(x,t\right)$. The magnitude of the strain energy *U* depends on the function $\:z\left(x,t\right)$.

**Fig. 4:**
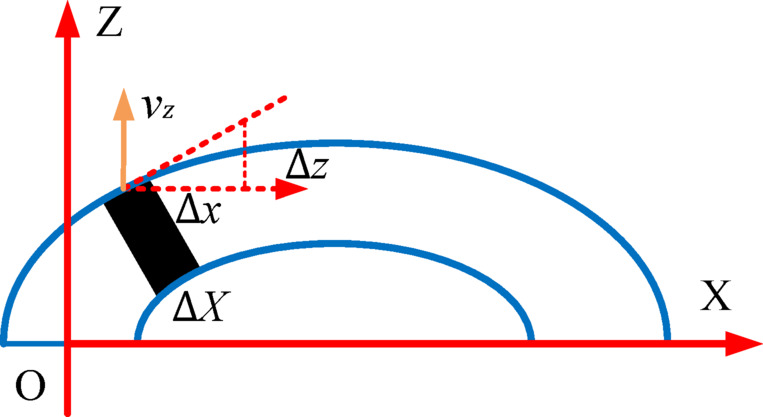
Diagram of slurry movement inside the pipe.

When slurry is conveyed through the slurry pipeline, the pressure disturbances from the discharge pump are transmitted through the flowing slurry to the pipe wall, causing a vertical acceleration, a Coriolis acceleration, and a curvature acceleration at any point along the pipeline’s central axis. The axial flow velocity of the slurry in the discharge pipeline is *v*, and the vertical velocity is $\:\frac{\partial\:z}{\partial\:t}$. When the pipeline bends due to disturbance pressure, the vertical component $\:{v}_{z}$ of the axial flow velocity is*v* shown in Fig. 4, and its calculation form is given by Formula (1).

1 $$\:{v}_{z}=\frac{{\Delta\:}z}{{\Delta\:}x}v=\frac{\partial\:z}{\partial\:x}v$$

Assuming the axial flow velocity *v* is constant.

At this moment, the speed in the z-direction is

2 $$\:\frac{\partial\:z}{\partial\:\mathrm{t}}+{v}_{z}=\frac{\partial\:z}{\partial\:\mathrm{t}}+\frac{\partial\:z}{\partial\:x}v$$

Considering the ∆*X* segment of the pipeline in Fig. 4, at this point, the kinetic energy of the fully loaded pipeline is the sum of the kinetic energy of this segment of the pipeline and the kinetic energy of the slurry inside the pipeline, that is

3 $$\:\frac{\mathrm{d}T}{\mathrm{d}x}=\frac{1}{2}\left(m-\rho\:\right){\left(\frac{\partial\:z}{\partial\:t}\right)}^{2}+\frac{1}{2}\rho\:\left[{{v}^{2}+\left(\frac{\partial\:\mathrm{z}}{\partial\:\mathrm{t}}+\frac{\partial\:\mathrm{z}}{\partial\:\mathrm{x}}v\right)}^{2}\right]$$

For ease of calculation and analysis, let $\:\dot{z}=\frac{\partial\:z}{\partial\:t},\:{\:z}^{{\prime\:}}=\frac{\partial\:z}{\partial\:x}$.

Under the influence of kinetic energy, the bent deformed slurry pipeline generates strain potential energy. For a continuous elastic body, the strain energy is related to the displacement of all points. Since there are infinitely many points, the displacement forms a curve and must be represented by a function. The strain energy U is a functional in the form of the size-dependent function z(*x*). The calculation method for strain potential energy can be derived from the Euler-Bernoulli beam theory with constant cross-section, as shown in Formula (4).

4 $$\:\mathrm{d}U=\frac{1}{2}EI{\left({z}^{{\prime\:}{\prime\:}}\right)}^{2}dx$$

Here, $\:I=\frac{\pi\:}{64}\left({D}^{4}-{d}^{4}\right)$ represents the moment of inertia for a circular cross-section pipe, where *D* is the outer diameter of the slurry conveying pipe and *d* is the inner diameter. *EI* = *k* denotes the flexural rigidity of the pipe.

To identify the true deflection state from all possible states allowed by constraints, based on the variational conditions satisfied by the real state (real displacements make the strain potential energy take an extremum, with the functional variation being zero), we derive formula (5) using the Hamiltonian variational principle of analytical mechanics and formulas (3) and (4).

5 $$\:{\updelta\:}{\int\:}_{{t}_{1}}^{{t}_{2}}{\int\:}_{{x}_{1}}^{{x}_{2}}\left\{\left[\frac{1}{2}\left(m-\rho\:\right){\left(\frac{\partial\:z}{\partial\:t}\right)}^{2}+\frac{1}{2}\rho\:\left[{{v}^{2}+\left(\frac{\partial\:\mathrm{z}}{\partial\:t}+\frac{\partial\:\mathrm{z}}{\partial\:x}v\right)}^{2}\right]\right]-\frac{1}{2}EI{\left({z}^{{\prime\:}{\prime\:}}\right)}^{2}\right\}dxdt=0$$

Where $\:{t}_{1}$ and $\:{t}_{2}$ represent the start and end times of the motion of the particle system, while $\:{x}_{1}$ and $\:{x}_{2}$ denote the initial and final positions of the motion, which are the constraint nodes.

In a complete physical system, the variational operation and the differential operation satisfy the following relationship,

6 $$\:\left\{\begin{array}{c}\delta\:\dot{z}=\:\frac{\partial\:}{\partial\:t}\left(\delta\:z\right)\\\:\delta\:{z}^{{\prime\:}}=\frac{\partial\:}{\partial\:x}\left(\delta\:z\right)\\\:\delta\:{z}^{{\prime\:}{\prime\:}}=\frac{{\partial\:}^{2}}{\partial\:{x}^{2}}\left(\delta\:z\right)\end{array}\right.$$

Using formula (6), after performing the variational operation on formula (5) and rearranging, we obtain formula (7),

7 $$\:{\int\:}_{{t}_{1}}^{{t}_{2}}{\int\:}_{{x}_{1}}^{{x}_{2}}\left\{\left(m-\rho\:\right)\dot{z}\delta\:\dot{z}+\rho\:\left(\dot{z}+v{z}^{{\prime\:}}\right)\left(\delta\:\dot{z}+v\delta\:{z}^{{\prime\:}}\right)-EI{z}^{{\prime\:}{\prime\:}}\delta\:{z}^{{\prime\:}{\prime\:}}\right\}dxdt=0$$

Expanding and rearranging formula (7) yields formula (8),

8 $$\:{\int\:}_{{t}_{1}}^{{t}_{2}}{\int\:}_{{x}_{1}}^{{x}_{2}}\left(m\dot{z}\delta\:\dot{z}+\rho\:v\dot{z}\delta\:{z}^{{\prime\:}}+\rho\:v{z}^{{\prime\:}}\delta\:\dot{z}+\rho\:{v}^{2}{z}^{{\prime\:}}\delta\:{z}^{{\prime\:}}-EI{z}^{{\prime\:}{\prime\:}}\delta\:{z}^{{\prime\:}{\prime\:}}\right)dxdt=0$$

To isolate the governing differential equation, we apply the method of integration by parts. This mathematical operation serves to transfer the time and spatial derivatives from the virtual displacement terms ($\:\delta\:\dot{z},\:\delta\:{z}^{{\prime\:}}$) to the state variables, thereby separating the domain equation from the boundary conditions.

The first product term inside the parentheses on the left side of Formula (8) yields Eqs. (9a) to (9c) after integration by parts,

9a $$\:{\int\:}_{{t}_{1}}^{{t}_{2}}{\int\:}_{{x}_{1}}^{{x}_{2}}m\dot{z}\delta\:\dot{z}dxdt={\int\:}_{{x}_{1}}^{{x}_{2}}{\int\:}_{{t}_{1}}^{{t}_{2}}m\dot{z}d\delta\:zdx$$

9b $$\:\begin{array}{c}{\int\:}_{{x}_{1}}^{{x}_{2}}{\int\:}_{{t}_{1}}^{{t}_{2}}m\dot{z}d\delta\:zdx={\int\:}_{{x}_{1}}^{{x}_{2}}\left[m\dot{z}\delta\:z\left|\begin{array}{c}{t}_{2}\\\:{t}_{1}\end{array}\right.-{\int\:}_{{t}_{1}}^{{t}_{2}}m\ddot{z}\delta\:zdt\right]dx\\\:\:\:\:\:\:\:\:\:\:\:\:\:\:\end{array}$$

9c $$\:{\int\:}_{{x}_{1}}^{{x}_{2}}\left[m\dot{z}\delta\:z\left|\begin{array}{c}{t}_{2}\\\:{t}_{1}\end{array}\right.-{\int\:}_{{t}_{1}}^{{t}_{2}}m\ddot{z}\delta\:zdt\right]dx=-{\int\:}_{{t}_{1}}^{{t}_{2}}{\int\:}_{{x}_{1}}^{{x}_{2}}m\ddot{z}\delta\:zdtdx$$

The second product term inside the parentheses on the left side of Formula (8) yields Eqs. (10a) to (10c) after integration by parts,

10a $$\:{\int\:}_{{t}_{1}}^{{t}_{2}}{\int\:}_{{x}_{1}}^{{x}_{2}}\rho\:v\dot{z}\delta\:{z}^{{\prime\:}}dxdt={\int\:}_{{t}_{1}}^{{t}_{2}}\left[{\int\:}_{{x}_{1}}^{{x}_{2}}\rho\:v\dot{z}d\delta\:z\right]dt$$

10b $$\:\begin{array}{c}{\int\:}_{{t}_{1}}^{{t}_{2}}\left[{\int\:}_{{x}_{1}}^{{x}_{2}}\rho\:v\dot{z}d\delta\:z\right]dt={\int\:}_{{t}_{1}}^{{t}_{2}}\left[\rho\:v\dot{z}\delta\:z\left|\begin{array}{c}{x}_{2}\\\:{x}_{1}\end{array}\right.-{\int\:}_{{x}_{1}}^{{x}_{2}}\rho\:v{\dot{z}}^{{\prime\:}}\delta\:zdx\right]dt\\\:\:\:\:\:\:\:\:\:\:\:\:\:\:\:\:\end{array}$$

10c $$\:{\int\:}_{{t}_{1}}^{{t}_{2}}\left[\rho\:v\dot{z}\delta\:z\left|\begin{array}{c}{x}_{2}\\\:{x}_{1}\end{array}\right.-{\int\:}_{{x}_{1}}^{{x}_{2}}\rho\:v{\dot{z}}^{{\prime\:}}\delta\:zdx\right]dt=-{\int\:}_{{t}_{1}}^{{t}_{2}}{\int\:}_{{x}_{1}}^{{x}_{2}}\rho\:v{\dot{z}}^{{\prime\:}}\delta\:zdxdt$$

The third product term inside the parentheses on the left side of Formula (8), after integration by parts, results in Eqs. (11a) to (11c),

11a $$\:{\int\:}_{{t}_{1}}^{{t}_{2}}{\int\:}_{{x}_{1}}^{{x}_{2}}\rho\:v{z}^{{\prime\:}}\delta\:\dot{z}dxdt={\int\:}_{{x}_{1}}^{{x}_{2}}{\int\:}_{{t}_{1}}^{{t}_{2}}\rho\:v{z}^{{\prime\:}}d\delta\:zdx$$

11b $$\:\begin{array}{c}{\int\:}_{{x}_{1}}^{{x}_{2}}{\int\:}_{{t}_{1}}^{{t}_{2}}\rho\:v{z}^{{\prime\:}}d\delta\:zdx={\int\:}_{{x}_{1}}^{{x}_{2}}\left[\rho\:v{z}^{{\prime\:}}\delta\:z\left|\begin{array}{c}{t}_{2}\\\:{t}_{1}\end{array}\right.-{\int\:}_{{t}_{1}}^{{t}_{2}}\rho\:v\dot{{z}^{{\prime\:}}}\delta\:zdt\right]dx\\\:\:\:\:\:\:\:\:\:\:\:\:\:\:\:\:\end{array}$$

11c $$\:{\int\:}_{{x}_{1}}^{{x}_{2}}\left[\rho\:v{z}^{{\prime\:}}\delta\:z\left|\begin{array}{c}{t}_{2}\\\:{t}_{1}\end{array}\right.-{\int\:}_{{t}_{1}}^{{t}_{2}}\rho\:v\dot{{z}^{{\prime\:}}}\delta\:zdt\right]dx=-{\int\:}_{{x}_{1}}^{{x}_{2}}{\int\:}_{{t}_{1}}^{{t}_{2}}\rho\:v\dot{{z}^{{\prime\:}}}\delta\:zdtdx$$

The fourth product term inside the parentheses on the left side of Formula (8), after integration by parts, results in Eqs. (12a) to (12c),

12a $$\:{\int\:}_{{t}_{1}}^{{t}_{2}}{\int\:}_{{x}_{1}}^{{x}_{2}}\rho\:{v}^{2}{z}^{{\prime\:}}\delta\:{z}^{{\prime\:}}dxdt={\int\:}_{{t}_{1}}^{{t}_{2}}{\int\:}_{{x}_{1}}^{{x}_{2}}\rho\:{v}^{2}{z}^{{\prime\:}}d\delta\:zdt$$

12b $$\:{\int\:}_{{t}_{1}}^{{t}_{2}}{\int\:}_{{x}_{1}}^{{x}_{2}}\rho\:{v}^{2}{z}^{{\prime\:}}d\delta\:zdt={\int\:}_{{t}_{1}}^{{t}_{2}}\left[\rho\:{v}^{2}{z}^{{\prime\:}}\delta\:z\left|\begin{array}{c}{x}_{2}\\\:{x}_{1}\end{array}\right.-{\int\:}_{{x}_{1}}^{{x}_{2}}\rho\:{v}^{2}{z}^{{\prime\:}{\prime\:}}\delta\:zdx\right]dt$$

12c $$\:{\int\:}_{{t}_{1}}^{{t}_{2}}\left[\rho\:{v}^{2}{z}^{{\prime\:}}\delta\:z\left|\begin{array}{c}{x}_{2}\\\:{x}_{1}\end{array}\right.-{\int\:}_{{x}_{1}}^{{x}_{2}}\rho\:{v}^{2}{z}^{{\prime\:}{\prime\:}}\delta\:zdx\right]dt=-{\int\:}_{{t}_{1}}^{{t}_{2}}{\int\:}_{{x}_{1}}^{{x}_{2}}\rho\:{v}^{2}{z}^{{\prime\:}{\prime\:}}\delta\:zdxdt$$

The fifth product term in the left parenthesis of Formula (8), after two-stage step-by-step integration, results in Eqs. (13a) to (13f),

13a $$\:{\int\:}_{{t}_{1}}^{{t}_{2}}{\int\:}_{{x}_{1}}^{{x}_{2}}EI{z}^{{\prime\:}{\prime\:}}\delta\:{z}^{{\prime\:}{\prime\:}}dxdt={\int\:}_{{t}_{1}}^{{t}_{2}}{\int\:}_{{x}_{1}}^{{x}_{2}}EI{z}^{{\prime\:}{\prime\:}}d\delta\:{z}^{{\prime\:}}dt$$

13b $$\:{\int\:}_{{t}_{1}}^{{t}_{2}}{\int\:}_{{x}_{1}}^{{x}_{2}}EI{z}^{{\prime\:}{\prime\:}}d\delta\:{z}^{{\prime\:}}dt={\int\:}_{{t}_{1}}^{{t}_{2}}\left[EI{z}^{{\prime\:}{\prime\:}}\delta\:{z}^{{\prime\:}}\left|\begin{array}{c}{x}_{2}\\\:{x}_{1}\end{array}-{\int\:}_{{x}_{1}}^{{x}_{2}}EI{z}^{\left(3\right)}\delta\:{z}^{{\prime\:}}dx\right.\right]dt$$

13c $$\:{\int\:}_{{t}_{1}}^{{t}_{2}}\left[EI{z}^{{\prime\:}{\prime\:}}\delta\:{z}^{{\prime\:}}\left|\begin{array}{c}{x}_{2}\\\:{x}_{1}\end{array}-{\int\:}_{{x}_{1}}^{{x}_{2}}EI{z}^{\left(3\right)}\delta\:{z}^{{\prime\:}}dx\right.\right]dt=-{\int\:}_{{t}_{1}}^{{t}_{2}}{\int\:}_{{x}_{1}}^{{x}_{2}}EI{z}^{\left(3\right)}\delta\:{z}^{{\prime\:}}dxdt$$

13d $$\:-{\int\:}_{{t}_{1}}^{{t}_{2}}{\int\:}_{{x}_{1}}^{{x}_{2}}EI{z}^{\left(3\right)}\delta\:{z}^{{\prime\:}}dxdt=-{\int\:}_{{t}_{1}}^{{t}_{2}}{\int\:}_{{x}_{1}}^{{x}_{2}}EI{z}^{\left(3\right)}d\delta\:zdt$$

13e $$\:-{\int\:}_{{t}_{1}}^{{t}_{2}}{\int\:}_{{x}_{1}}^{{x}_{2}}EI{z}^{\left(3\right)}d\delta\:zdt=-{\int\:}_{{t}_{1}}^{{t}_{2}}\left[EI{z}^{\left(3\right)}\delta\:z\left|\begin{array}{c}{x}_{2}\\\:{x}_{1}\end{array}-{\int\:}_{{x}_{1}}^{{x}_{2}}EI{z}^{\left(4\right)}\delta\:zdx\right.\right]dt$$

13f $$\:-{\int\:}_{{t}_{1}}^{{t}_{2}}\left[EI{z}^{\left(3\right)}\delta\:z\left|\begin{array}{c}{x}_{2}\\\:{x}_{1}\end{array}-{\int\:}_{{x}_{1}}^{{x}_{2}}EI{z}^{\left(4\right)}\delta\:zdx\right.\right]dt={\int\:}_{{t}_{1}}^{{t}_{2}}{\int\:}_{{x}_{1}}^{{x}_{2}}EI{z}^{\left(4\right)}\delta\:zdxdt$$

By substituting the integrated terms back into the variational equation and assuming that the boundary terms vanish due to the fixed supports, we collect all terms associated with the arbitrary virtual displacement $\:\delta\:z$ inside the time-space integral.

From Eqs. (8), (9c) to (12c), and Formula (13f), we obtain Formula (14),

14 $$\:{\int\:}_{{t}_{1}}^{{t}_{2}}{\int\:}_{{x}_{1}}^{{x}_{2}}\left(-m\ddot{z}-2\rho\:v{\dot{z}}^{{\prime\:}}-\rho\:{v}^{2}{z}^{{\prime\:}{\prime\:}}-EI{z}^{\left(4\right)}\right)\delta\:zdxdt=0$$

According to the Fundamental Lemma of Calculus of Variations, since the variation $\:\delta\:z$ is arbitrary within the domain, the integrand itself must be identically zero to satisfy the equation. This yields the final governing partial differential equation for the slurry pipeline:

15 $$\:\mathrm{E}\mathrm{I}\frac{{\partial\:}^{4}\mathrm{z}}{\partial\:{x}^{4}}+\rho\:{v}^{2}\frac{{\partial\:}^{2}\mathrm{z}}{\partial\:{x}^{2}}+m\frac{{\partial\:}^{2}\mathrm{z}}{\partial\:{t}^{2}}=-2\rho\:v\frac{{\partial\:}^{2}z}{\partial\:x\partial\:t}$$

Physically, as the pipe vibrates, any segment of the pipe rotates with an angular velocity locally approximated by the rate of change of the slope ($\:\frac{\partial\:}{\partial\:t}\left(\frac{\partial\:z}{\partial\:x}\right)$). As the fluid element moves with velocity *v* along this rotating path, it experiences a Coriolis-like acceleration. This interpretation is standard in classical literature on fluid-structure interaction in pipes (e.g., Païdoussis, M. P.,1998, “Fluid-Structure Interactions: Slender Structures and Axial Flow"^27^).

The above analytical expression clearly demonstrates the physical effects of each term. In the equation, $\:\mathrm{E}\mathrm{I}\frac{{\partial\:}^{4}z}{\partial\:{x}^{4}}$ represents the bending stiffness of the slurry pipeline, $\:\rho\:{v}^{2}\frac{{\partial\:}^{2}\mathrm{z}}{\partial\:{x}^{2}}$ represents the centrifugal inertial force caused by slurry flow, $\:m\frac{{\partial\:}^{2}\mathrm{z}}{\partial\:{t}^{2}}$ represents the vertical inertial force of the pipeline mass, and $\:2\rho\:v\frac{{\partial\:}^{2}z}{\partial\:x\partial\:t}$ represents the dynamic coupling term due to the Coriolis force, which signifies the Coriolis acceleration and curvature effect, resulting from the coupling of slurry flow velocity and pipeline vibration velocity.

## Solving and verification

### Solving model equations

To derive the analytical expression (15) for the dynamic differential equation of deflection z for a slurry shield machine single-span conveying pipe when fully loaded, it is rewritten in the form of Formula (16a).

16a $$\:\mathrm{E}\mathrm{I}\frac{{\partial\:}^{4}\mathrm{z}}{\partial\:{x}^{4}}+{\uprho\:}{\mathrm{v}}^{2}\frac{{\partial\:}^{2}\mathrm{z}}{\partial\:{x}^{2}}+\mathrm{m}\frac{{\partial\:}^{2}\mathrm{z}}{\partial\:{t}^{2}}=\mathrm{F}\left(x,t\right)$$

When $\:F\left(x,t\right)=0$, Formula (16b) becomes a homogeneous constant coefficient differential equation of Formula (16a).

16b $$\:\mathrm{E}\mathrm{I}\frac{{\partial\:}^{4}\mathrm{z}}{\partial\:{x}^{4}}+\rho\:{v}^{2}\frac{{\partial\:}^{2}\mathrm{z}}{\partial\:{x}^{2}}+m\frac{{\partial\:}^{2}\mathrm{z}}{\partial\:{t}^{2}}=0$$

Using the method of separation of variables, set the form of the solution as,

17 $$\:\mathrm{z}\left(x,t\right)=X\left(x\right)T\left(t\right)$$

Here, $\:X\left(x\right)$ is a function of the spatial variable x, and $\:T\left(t\right)$ is a function of the time variable t.

Substituting $\:\mathrm{z}\left(x,t\right)=X\left(x\right)T\left(t\right)$ into Formula (16b), we obtain

18 $$\:\mathrm{E}\mathrm{I}{X}^{\left(4\right)}\left(x\right)T\left(t\right)+\rho\:{v}^{2}{X}^{{\prime\:}{\prime\:}}\left(x\right)T\left(t\right)+mX\left(x\right){T}^{{\prime\:}{\prime\:}}\left(t\right)=0$$

Dividing both sides by $\:X\left(x\right)T\left(t\right)$ and rearranging, we get

19 $$\:\mathrm{E}\mathrm{I}\frac{{\mathrm{X}}^{\left(4\right)}\left(x\right)}{X\left(x\right)}+\rho\:{v}^{2}\frac{{X}^{{\prime\:}{\prime\:}}\left(x\right)}{X\left(x\right)}=-m\frac{{T}^{{\prime\:}{\prime\:}}\left(t\right)}{T\left(t\right)}$$

Since the left side of Formula (19) is an expression involving *x*, and the right side involves *t*, for Formula (19) to hold true, both sides must equal a constant. Let this constant be λ, resulting in the following two independent equations, namely Eqs. (20) and (21a),

20 $$\:-m\frac{{T}^{{\prime\:}{\prime\:}}\left(t\right)}{T\left(t\right)}={\uplambda\:}$$

21a $$\:\mathrm{E}\mathrm{I}\frac{{\mathrm{X}}^{\left(4\right)}\left(x\right)}{X\left(x\right)}+\rho\:{v}^{2}\frac{{X}^{{\prime\:}{\prime\:}}\left(x\right)}{X\left(x\right)}={\uplambda\:}$$

Solve for $\:X\left(x\right)$ in the equation.

Considering the boundary conditions$\:\:\mathrm{z}\left(0,t\right)=\mathrm{z}\left(L,t\right)=0$, let $\:X\left(x\right)=\mathrm{sin}\left(kx\right)$ or $\:X\left(x\right)=\mathrm{cos}\left(kx\right)$, where *L* is the axial length of the single-span discharge pipeline when it is not bent, and *k* is a constant. According to the boundary conditions, the function is zero at x=0 or x=L, so kL=nπ, $\:k=\frac{n\pi\:}{L}$, where,

21b $$\:{\mathrm{X}}_{n}\left(x\right)=\mathrm{sin}\left(\frac{n\pi\:}{L}x\right)$$

where n is a non-zero positive integer.

Substituting Formula (21b) into the differential Formula (21a) yields

22 $$\:\mathrm{E}\mathrm{I}{\left(\frac{n\pi\:}{L}\right)}^{4}\mathrm{sin}\left(\frac{n\pi\:x}{L}\right)-\rho\:{v}^{2}{\left(\frac{n\pi\:}{L}\right)}^{2}\mathrm{s}\mathrm{i}\mathrm{n}\left(\frac{n\pi\:x}{L}\right)-{\uplambda\:}\mathrm{s}\mathrm{i}\mathrm{n}\left(\frac{n\pi\:x}{L}\right)=0$$

After organizing, we get

23 $$\:{\lambda\:}_{n}=\mathrm{E}\mathrm{I}{\left(\frac{n\pi\:}{L}\right)}^{4}-\rho\:{v}^{2}{\left(\frac{n\pi\:}{L}\right)}^{2}$$

Solve for $\:T\left(t\right)$ in the equation.

Using formula (20) in conjunction with formula (23), we can derive formula (24).

24 $$\:m\frac{{d}^{2}T}{d{t}^{2}}+{\lambda\:}_{n}T=0$$

The solution is formula (25).

25 $$\:{T}_{n}={A}_{n}{sin}\left(\sqrt{\frac{{\lambda\:}_{n}}{m}}t\right)+{B}_{n}{cos}\left(\sqrt{\frac{{\lambda\:}_{n}}{m}}t\right)$$

Here, A_n_ and B_n_ are constants.

By taking into account the solutions for $\:X\left(x\right)$ and $\:T\left(t\right)$, i.e., Eqs. (21b) and (25), we can derive the analytical expressions (26a) and (26b) for Formula (16b).

26a $$\:z\left(x,t\right)=\sum\:_{n=1}^{\infty\:}{sin}\left(\frac{n\pi\:x}{L}\right)\left[{A}_{n}{sin}\left({\omega\:}_{n}t\right)+{B}_{n}{cos}\left({\omega\:}_{n}t\right)\right]$$

26b $$\:{z}_{n}\left(x,t\right)={sin}\left(\frac{n\pi\:x}{L}\right)\left[{A}_{n}{sin}\left({\omega\:}_{n}t\right)+{B}_{n}{cos}\left({\omega\:}_{n}t\right)\right]$$

Where $\:{\omega\:}_{n}=\sqrt{\frac{{\lambda\:}_{n}}{m}}=\left(\frac{n\pi\:}{L}\right)\sqrt{\frac{EI}{m}{\left(\frac{n\pi\:}{L}\right)}^{2}-\frac{\rho\:{v}^{2}}{m}}$. This equation shows that when the slurry fluid is stationary (*v* = 0), $\:{\omega\:}_{n}\propto\:{n}^{2}$, which corresponds to the classical Euler-Bernoulli beam frequency. At this point, the natural angular frequency of the pipeline, i.e., the frequency during free vibration, is given by $\:{\omega\:}_{n}={(\mathrm{n}{\uppi\:}/\mathrm{L})}^{2}\sqrt{\frac{EI}{m}}\:$, where *n* is a positive integer. When slurry fluid flows (*v* > 0), it has an effect of reducing the inherent frequency of the pipeline. When $\:\rho\:\mathrm{*}{v}^{2}\ge\:EI{\left(\frac{n\mathrm{*}\pi\:}{L}\right)}^{2}$, complex frequencies occur, indicating that the system is in an unstable state.

When $\:F\left(x,t\right)=-2\rho\:v\frac{{\partial\:}^{2}z}{\partial\:x\partial\:t}$, Eqs. (27a) and (27b) can be derived.

27a $$\:F\left(x,t\right)=-2\rho\:v\sum\:_{n=1}^{\infty\:}\left(\frac{{n\pi\:}}{L}\right){cos}\left(\frac{n\pi\:x}{L}\right)\left[{A}_{n}{\omega\:}_{n}{cos}\left({\omega\:}_{n}t\right)-{B}_{n}{\omega\:}_{n}{sin}\left({\omega\:}_{n}t\right)\right]$$

27b $$\:{F}_{n}\left(x,t\right)=-2{\rho\:v}\left(\frac{{n\pi\:}}{L}\right){cos}\left(\frac{n\pi\:x}{L}\right)\left[{A}_{n}{\omega\:}_{n}{cos}\left({\omega\:}_{n}t\right)-{B}_{n}{\omega\:}_{n}{sin}\left({\omega\:}_{n}t\right)\right]$$

By comparing the expressions containing the spatial variable *x* in formulas (26b) and (27b), it can be observed that there is a 90° phase difference between $\:{F}_{n}$ and the corresponding displacement $\:{z}_{n}$ during free vibration. The vibration mode shape $\:{\mathrm{z}}_{n}$ of a single-span pipe during free vibration is symmetric with respect to the midpoint, while $\:{F}_{n}$ should be the corresponding antisymmetric resonance mode. The appearance of $\:{F}_{n}$ will trigger all asymmetric modes, and any one of these asymmetric modes will generate an external force $\:{F}_{n}$ that excites all symmetric modes. Therefore, the solution of Formula (15) should be a linear combination of all modes in Formula (16a) where $\:F\left(x,t\right)=-2\rho\:v\frac{{\partial\:}^{2}z}{\partial\:x\partial\:t}$, with the symmetric mode being 90° out of phase with the asymmetric mode. The second-order mixed derivative term $\:\frac{{\partial\:}^{2}z}{\partial\:x\partial\:t}$ achieves the dynamic coupling of symmetric and asymmetric modes. Considering the odd and even properties and periodicity of the sine function, odd modes typically represent symmetric vibrations (with the pipe’s support end as a node), while even modes describe asymmetric modes (with the midpoint of the single-span pipe as a node). By decomposing the vibration modes into odd and even parts, we can more clearly analyze and understand the dynamic behavior of the slurry shield muck discharge pipeline, especially when the pipeline system is subjected to specific types of external forces (such as$\:\:F\left(x,t\right)=-2\rho\:v\frac{{\partial\:}^{2}z}{\partial\:x\partial\:t}$). Therefore, the solution for $\:\mathrm{z}\left(x,t\right)$ in Formula (15) can be described using Eqs. (28a) and (28b).

28a $$\:z\left(x,t\right)=\sum\:_{n=1}^{\infty\:}\left[\sum\:_{j=1}^{\infty\:}{A}_{2j-1}{sin}\left(\left(2j-1\right)\frac{\pi\:x}{L}\right){sin}\left({\omega\:}_{n}t\right)+\sum\:_{k=1}^{\infty\:}{A}_{2k}{sin}\left(2k\frac{\pi\:x}{L}\right){cos}\left({\omega\:}_{n}t\right)\right]$$

28b $$\:{\mathrm{z}}_{n}\left(x,t\right)=\sum\:_{j=1}^{{\infty\:}}{A}_{2j-1}\mathrm{sin}\left(\left(2j-1\right)\frac{\pi\:x}{L}\right)\mathrm{sin}\left({\omega\:}_{n}t\right)+\sum\:_{k=1}^{{\infty\:}}{A}_{2k}\mathrm{sin}\left(2k\frac{\pi\:x}{L}\right)\mathrm{cos}\left({\omega\:}_{n}t\right)$$

Here, the symmetric mode is a combination of odd harmonic $\:\left(\mathrm{sin}\left(\left(2j-1\right)\frac{\pi\:x}{L}\right)\right)$ with the time term $\:\mathrm{sin}\left({\omega\:}_{n}t\right)$; the antisymmetric mode consists of even harmonic $\:\left(\mathrm{sin}\left(2k\frac{\pi\:x}{L}\right)\right)$ combined with the time term $\:\mathrm{cos}\left({\omega\:}_{n}t\right)$, leading the symmetric mode by 90 degrees in phase.

This expression indicates that the structure of a single-mode $\:{\mathrm{z}}_{n}$ includes spatial harmonic terms of odd and even orders, with different phase time dependencies $\:\mathrm{sin}\left({\omega\:}_{n}\right)$ and $\:\mathrm{cos}\left({\omega\:}_{n}\right)$, resulting in mode shapes in complex form. The overall solution structure, i.e., the deflection displacement $\:\mathrm{z}(x,t)$, is a linear superposition of all modes $\:{\mathrm{z}}_{n}$.

In solving the equation for natural frequencies, each mode shape $\:{\mathrm{z}}_{n}$ corresponds to a natural frequency $\:{\omega\:}_{n}$. However, due to the coupling relationship between odd and even terms, the frequency equation must consider multiple harmonic terms simultaneously, resulting in each $\:{\omega\:}_{n}$ being the effect of multiple harmonics acting together.

Substituting Formula (28a) into Formula (15), we can calculate each derivative term of the differential equation to obtain formulas (29a) to (29d).

29a $$\:\frac{{\partial\:}^{\left(4\right)}\mathrm{z}}{\partial\:{x}^{4}}=\sum\:_{n=1}^{{\infty\:}}\left[\sum\:_{j=1}^{{\infty\:}}{\mathrm{A}}_{2j-1}{\left(2j-1\right)}^{4}{\left(\frac{{\uppi\:}}{L}\right)}^{4}\mathrm{sin}\left(\left(2j-1\right)\frac{{\uppi\:}x}{L}\right)\mathrm{sin}\left({\omega\:}_{n}t\right)+\sum\:_{k=1}^{{\infty\:}}{\mathrm{A}}_{2k}{\left(2k\right)}^{4}{\left(\frac{{\uppi\:}}{L}\right)}^{4}\mathrm{sin}\left(2k\frac{{\uppi\:}x}{L}\right)\mathrm{cos}\left({\omega\:}_{n}t\right)\right]$$

29b $$\:\frac{{\partial\:}^{\left(2\right)}\mathrm{z}}{\partial\:{x}^{2}}=\sum\:_{n=1}^{{\infty\:}}\left[\sum\:_{j=1}^{{\infty\:}}{{{-\mathrm{A}}_{2j-1}\left(2j-1\right)}^{2}\left(\frac{{\uppi\:}}{L}\right)}^{2}\mathrm{sin}\left(\left(2j-1\right)\frac{{\uppi\:}x}{L}\right)\mathrm{sin}\left({\omega\:}_{n}t\right)+\sum\:_{k=1}^{{\infty\:}}{-\mathrm{A}}_{2k}{\left(2k\right)}^{2}{\left(\frac{{\uppi\:}}{L}\right)}^{2}\mathrm{sin}\left(2k\frac{{\uppi\:}x}{L}\right)\mathrm{cos}\left({\omega\:}_{n}t\right)\right]$$

29c $$\:\frac{{\partial\:}^{\left(2\right)}\mathrm{z}}{\partial\:{t}^{2}}=\sum\:_{n=1}^{{\infty\:}}\left[\sum\:_{j=1}^{{\infty\:}}{A}_{2j-1}(-{\omega\:}_{n}^{2})\mathrm{sin}\left(\left(2j-1\right)\frac{\pi\:x}{L}\right)\mathrm{sin}\left({\omega\:}_{n}t\right)+\sum\:_{k=1}^{{\infty\:}}{A}_{2k}(-{\omega\:}_{n}^{2})\mathrm{sin}\left(2k\frac{\pi\:x}{L}\right)\mathrm{cos}\left({\omega\:}_{n}t\right)\right]$$

29d $$\:\frac{{\partial\:}^{\left(2\right)}z}{\partial\:x\partial\:t}=\sum\:_{n=1}^{{\infty\:}}\left[-\sum\:_{k=1}^{{\infty\:}}{A}_{2k}\left(2k\right)\left(\frac{\pi\:}{L}\right)\left({\omega\:}_{n}\right){cos}\left(2k\frac{\pi\:x}{L}\right){sin}\left({\omega\:}_{n}t\right)+\sum\:_{j=1}^{{\infty\:}}{A}_{2j-1}\left(2j-1\right)\left(\frac{\pi\:}{L}\right)\left({\omega\:}_{n}\right){cos}\left(\left(2j-1\right)\frac{\pi\:x}{L}\right){cos}\left({\omega\:}_{n}t\right)\right]$$

By examining the right-hand side of Eqs. (29a) to (29d), which contain terms like $\:\mathrm{s}\mathrm{i}\mathrm{n}\left({\omega\:}_{n}t\right)$ and $\:\mathrm{c}\mathrm{o}\mathrm{s}\left({\omega\:}_{n}t\right)$, it is evident that to combine like terms, the cosine term $\:\mathrm{cos}\left(2k\frac{{\uppi\:}x}{L}\right)$ in Formula (29d) needs to be approximated by a series of sine functions, specifically $\:\mathrm{sin}\left(\left(2j-1\right)\frac{\pi\:x}{L}\right)$. Similarly, $\:\mathrm{cos}\left(\left(2j-1\right)\frac{{\uppi\:}x}{L}\right)$ must be approximated by a series of sine functions of $\:\mathrm{sin}\left(2k\frac{\pi\:x}{L}\right)$.

Since the functions $\:\mathrm{cos}\left(2k\frac{{\uppi\:}x}{L}\right)$ and $\:\mathrm{cos}\left(\left(2j-1\right)\frac{{\uppi\:}x}{L}\right)$ need to be approximated by the Fourier series of $\:\mathrm{sin}\left(\left(2j-1\right)\frac{{\uppi\:}x}{L}\right)$ and $\:\mathrm{sin}\left(2k\frac{{\uppi\:}x}{L}\right)$ respectively, odd extensions of the functions $\:\mathrm{cos}\left(2k\frac{{\uppi\:}x}{L}\right)$ and $\:\mathrm{cos}\left(\left(2j-1\right)\frac{{\uppi\:}x}{L}\right)$ are required.

The detailed derivation of the Fourier series coefficients for the odd and even extensions is provided in Appendix A. Using the coefficients derived in Appendix A (specifically Eqs. A19 and A27), and substituting the series expansions back into the governing equation, we obtain the coupled expression.

Combining formulas (29d), (A2), (A19), (A20), and (A27) results in formula (30).

30 $$\:\frac{{\partial\:}^{\left(2\right)}z}{\partial\:x\partial\:t}=\sum\:_{n=1}^{{\infty\:}}\left[\begin{array}{c}-\sum\:_{k=1}^{{\infty\:}}{A}_{2k}\left(2k\right)\left(\frac{\pi\:}{L}\right)\left({\omega\:}_{n}\right)\left(\sum\:_{j=1}^{{\infty\:}}\left(\frac{4}{\pi\:}\frac{\left(2j-1\right)}{{\left(2j-1\right)}^{2}-{\left(2k\right)}^{2}}\right)\mathrm{sin}\left(\left(2j-1\right)\frac{\pi\:x}{L}\right)\right)\mathrm{sin}\left({\omega\:}_{n}t\right)\:\\\:+\sum\:_{j=1}^{{\infty\:}}{A}_{2j-1}\left(2j-1\right)\left(\frac{\pi\:}{L}\right)\left({\omega\:}_{n}\right)\left(\sum\:_{k=1}^{{\infty\:}}\left(\frac{4}{\pi\:}\frac{\left(2k\right)}{{\left(2k\right)}^{2}-{\left(2j-1\right)}^{2}}\right)\mathrm{sin}\left(2k\frac{\pi\:x}{L}\right)\right)\mathrm{cos}\left({\omega\:}_{n}t\right)\end{array}\right]$$

After simplification and rearrangement, formula (30) can be transformed into formula (31).

31 $$\:\frac{{\partial\:}^{\left(2\right)}z}{\partial\:x\partial\:t}=\sum\:_{n=1}^{\infty\:}\left[\begin{array}{c}-\sum\:_{j=1}^{\infty\:}\sum\:_{k=1}^{\infty\:}{A}_{2k}\left(2k\right)\left(\frac{4}{L}\right)\left({\omega\:}_{n}\right)\left(\frac{\left(2j-1\right)}{{\left(2j-1\right)}^{2}-{\left(2k\right)}^{2}}\right){sin}\left(\left(2j-1\right)\frac{\pi\:x}{L}\right){sin}\left({\omega\:}_{n}t\right)\\\:+\sum\:_{k=1}^{\infty\:}\sum\:_{j=1}^{\infty\:}{A}_{2j-1}\left(2j-1\right)\left(\frac{4}{L}\right)\left({\omega\:}_{n}\right)\left(\frac{\left(2k\right)}{{\left(2k\right)}^{2}-{\left(2j-1\right)}^{2}}\right){sin}\left(2k\frac{{\pi\:x}}{L}\right){cos}\left({\omega\:}_{n}t\right)\end{array}\right]$$

Substitute Eqs. (29a) to (29c) and Formula (31) into Formula (15), combine like terms, set the coefficient of the term $\:\mathrm{sin}\left(\left(2j-1\right)\frac{{\uppi\:}x}{L}\right)\mathrm{sin}\left({\omega\:}_{n}t\right)$ to zero, resulting in Formula (32).

32 $$\:{A}_{2j-1}\left[EI{\left(2j-1\right)}^{4}{\left(\frac{\pi\:}{L}\right)}^{4}-\rho\:{v}^{2}{\left(2j-1\right)}^{2}{\left(\frac{\pi\:}{L}\right)}^{2}-m{{\omega\:}_{n}}^{2}\right]=2\rho\:v\sum\:_{k=1}^{\infty\:}{A}_{2k}\left(2k\right)\left(\frac{4}{L}\right)\left({\omega\:}_{n}\right)\left(\frac{\left(2j-1\right)}{{\left(2j-1\right)}^{2}-{\left(2k\right)}^{2}}\right)$$

After rearranging formula (32), we obtain formula (33).

33 $$\:{A}_{2j-1}\left[EI{\left(2j-1\right)}^{4}{\left(\frac{\pi\:}{L}\right)}^{4}-\rho\:{v}^{2}{\left(2j-1\right)}^{2}{\left(\frac{\pi\:}{L}\right)}^{2}-m{{\omega\:}_{n}}^{2}\right]=\left(\frac{8\rho\:v{\omega\:}_{n}}{L}\right)\sum\:_{k=1}^{\infty\:}{A}_{2k}\left(\frac{\left(2k\right)\left(2j-1\right)}{{\left(2j-1\right)}^{2}-{\left(2k\right)}^{2}}\right)$$

Setting the coefficient of the like term $\:\mathrm{sin}\left(2\mathrm{k}\frac{{\uppi\:}\mathrm{x}}{\mathrm{L}}\right)\mathrm{cos}\left({{\upomega\:}}_{\mathrm{n}}\mathrm{t}\right)$ to zero yields Formula (34).

34 $$\:{A}_{2k}\left[EI{\left(2k\right)}^{4}{\left(\frac{\pi\:}{L}\right)}^{4}-\rho\:{v}^{2}{\left(2k\right)}^{2}{\left(\frac{\pi\:}{L}\right)}^{2}-m{{\omega\:}_{n}}^{2}\right]=-2\rho\:v\sum\:_{j=1}^{\infty\:}{A}_{2j-1}\left(2j-1\right)\left(\frac{4}{L}\right)\left({\omega\:}_{n}\right)\left(\frac{\left(2k\right)}{{\left(2k\right)}^{2}-{\left(2j-1\right)}^{2}}\right)$$

After rearranging formula (34), we obtain formula (35).

35 $$\:{A}_{2k}\left[EI{\left(2k\right)}^{4}{\left(\frac{\pi\:}{L}\right)}^{4}-\rho\:{v}^{2}{\left(2k\right)}^{2}{\left(\frac{\pi\:}{L}\right)}^{2}-m{{\omega\:}_{n}}^{2}\right]=-\left(\frac{8\rho\:v{\omega\:}_{n}}{L}\right)\sum\:_{j=1}^{\infty\:}{A}_{2j-1}\left(\frac{\left(2k\right)\left(2j-1\right)}{{\left(2k\right)}^{2}-{\left(2j-1\right)}^{2}}\right)$$

Equations (33) and (35) reveal the dynamic coupling between odd mode shapes (symmetrical vibration) and even mode shapes (asymmetrical vibration) in the vibration of the slurry pipeline. The amplitude $\:{A}_{2j-1}$ of any odd mode is affected by the amplitudes of all even modes (Formula 33), and conversely, the amplitude $\:{A}_{2k}$ of any even mode is influenced by the amplitudes of all odd modes (Formula 35). This coupling effect is driven by the flow velocity *v* of the slurry and the Coriolis force term.

### Engineering applications

In practice, higher-order terms must be truncated (such as retaining the first N terms) to convert the infinite-dimensional system of equations into a finite-dimensional matrix equation. After truncation to Nth order, the odd (Formula 33) and even (Formula 35) mode coupling equations can be represented as a $\:2N\times\:2N$ matrix equation, as shown in Formula (36).

36 $$\:K\left({\omega\:}_{n}\right)A=0$$

Where the diagonal elements represent mode self-coupling terms (bending stiffness, centrifugal force, mass inertia terms) and the off-diagonal elements represent dynamic coupling terms between odd and even modes (including flow velocity *v* and Coriolis force term).

Using the determinant condition shown in Formula (37), the natural frequencies $\:{\omega\:}_{n}$ of each order are obtained by solving the equation numerically. In this study, the symbolic derivation of the matrix elements and the numerical identification of the roots for the transcendental determinant equation were implemented in the MATLAB (R2021b) environment, utilizing the symbolic math toolbox and the fzero numerical solver to ensure computational precision.

37 $$\:{det}\left(K\left({\omega\:}_{n}\right)\right)=0$$

For each $\:{\omega\:}_{n}$, substitute into the matrix equation and set the reference amplitude (such as $\:{\mathrm{A}}_{1}=1$), solve the linear equation system to obtain the odd-mode amplitudes $\:{\mathrm{A}}_{2j-1}$ (symmetric modes) and even-mode amplitudes $\:{\mathrm{A}}_{2k}$ (antisymmetric modes).

For ease of description, Formula (33) can be simplified to be (A_2j−1_) * [Odd − order mode self − coupling term] = ∑_k_ (Coupling term with even − order mode) * A_2k_; for Formula (35), (A_2k_) * [Even − order mode self − coupling term] = ∑_j_ (Coupling term with odd − order mode) * A_2j−1_.

This indicates that the equation for each odd-mode (Formula 33) involves only its own self-coupling term and coupling terms with even modes, while the equation for each even-mode (Formula 35) includes only its own self-coupling term and coupling terms with odd modes. Therefore, the equations for odd-modes should be placed in odd rows, and the equations for even-modes should be placed in even rows.

The right side of Formula (33) contains only the amplitudes of even mode orders. From the structure of this formula, it can be seen that odd mode orders couple only with even mode orders and are unrelated to other odd mode orders. Therefore, the elements in the coefficient matrix $\:K\left({\omega\:}_{n}\right)$ between odd-mode rows and odd-mode columns should be zero, and only the elements in positions corresponding to even-mode columns should be non-zero. Similarly, based on formula (35), even-order modes couple only with odd-order modes and are unrelated to other even-order modes. Therefore, the elements in the coefficient matrix $\:K\left({\omega\:}_{n}\right)$ between rows of even-order modes and other even-order columns should be zero, while non-zero elements appear only at positions corresponding to odd-order columns.

#### Definition of elements in the coefficient matrix $\:\boldsymbol{K}\left({\boldsymbol{\omega\:}}_{\boldsymbol{n}}\right)$

Mechanism of Matrix Construction:

The coefficient matrix $\:K\left({\omega\:}_{n}\right)$ is assembled by rewriting the coupled differential Eqs. (33) and (35) into a linear algebraic system $\:K\left({\omega\:}_{N}\right)\times\:A=0$.

The mapping rules are defined as follows:

1. Odd rows of the matrix (Row 2*j* − 1) correspond to Eq. (33). In these rows, the diagonal elements (Odd columns) are calculated using Eq. (38), and the coupling elements (Even columns) are calculated using Eq. (39).
2. Even rows of the matrix (Row 2*k*) correspond to Eq. (35). In these rows, the diagonal elements (Even columns) are calculated using Eq. (40), and the coupling elements (Odd columns) are calculated using Eq. (41).
3. Due to the specific coupling nature of the Coriolis force, odd modes only couple with even modes, resulting in zero values for elements $\:{K}_{odd,{odd}^{{\prime\:}}}$ (where *odd*$\:\ne\:$*odd*′) and $\:{K}_{even,{even}^{{\prime\:}}}$ (where *even*$\:\ne\:$*even*′).

For odd rows (odd-mode rows), the diagonal elements (self-coupling terms of the odd modes) are determined by Formula (38).

38 $$\:{K}_{2j-\mathrm{1,2}j-1}=EI{\left(2j-1\right)}^{4}{\left(\frac{\pi\:}{L}\right)}^{4}-\rho\:{v}^{2}{\left(2j-1\right)}^{2}{\left(\frac{\pi\:}{L}\right)}^{2}-m{\omega\:}_{n}^{2},\:(j=\mathrm{1,2},\dots\:,N)$$

The elements in the even columns of the row, i.e., the coupling terms with even-order modes, are determined by Formula (39).

39 $$\:{K}_{2j-\mathrm{1,2}k}=-\frac{8\rho\:v{\omega\:}_{n}}{L}\times\:\frac{\left(2k\right)\left(2j-1\right)}{{\left(2j-1\right)}^{2}-{\left(2k\right)}^{2}},(j=\mathrm{1,2},\dots\:,N;k=\mathrm{1,2},\dots\:,N)$$

The elements in the odd columns of the row are zero (independent of other odd-order modes).

For even rows (even mode rows), the diagonal elements (self-coupling terms of even modes) are determined by Formula (40).

40 $$\:{K}_{2k,2k}=EI{\left(2k\right)}^{4}{\left(\frac{\pi\:}{L}\right)}^{4}-\rho\:{v}^{2}{\left(2k\right)}^{2}{\left(\frac{\pi\:}{L}\right)}^{2}-m{\omega\:}_{n}^{2},(k=\mathrm{1,2},\dots\:,N)$$

The elements in the odd columns of the row, i.e., the coupling terms with odd-order modes, are determined by Formula (41).

41 $$\:{K}_{2k,2j-1}=\frac{8\rho\:v{\omega\:}_{n}}{L}\times\:\frac{\left(2k\right)(2j-1)}{{\left(2k\right)}^{2}-{(2j-1)}^{2}},(k=\mathrm{1,2},\dots\:,N；j=\mathrm{1,2},\dots\:,N)$$

The elements in the even columns of this row are zero (independent of other even-order modes). The assembly of the coefficient matrix *K*(*ω*_*n*_) follows a strict parity-based coupling logic. Odd rows of the matrix (2*j* − 1) correspond to Eq. (33), while even rows (2*k*) correspond to Eq. (35). A distinct feature of this matrix is its ‘checkerboard’ structure: because the Coriolis force only induces dynamic coupling between symmetric (odd) and asymmetric (even) vibration modes, all elements representing interactions between modes of the same parity (i.e., odd-odd or even-even) are zero. For *N* = 2, this results in a 4 × 4 matrix where *K*_1,3_=0 and *K*_2,4_=0, as shown below:

The matrix form for the example (*N* = 2 truncation) is,

$$\:\mathrm{K}=\left[\begin{array}{cc}\begin{array}{cc}{K}_{\mathrm{1,1}}&\:{K}_{\mathrm{1,2}}\\\:{K}_{\mathrm{2,1}}&\:{K}_{\mathrm{2,2}}\end{array}&\:\begin{array}{cc}0&\:{K}_{\mathrm{1,4}}\\\:{K}_{\mathrm{2,3}}&\:0\end{array}\\\:\begin{array}{cc}0&\:{K}_{\mathrm{3,2}}\\\:{K}_{\mathrm{4,1}}&\:0\end{array}&\:\begin{array}{cc}{K}_{\mathrm{3,3}}&\:{K}_{\mathrm{3,4}}\\\:{K}_{\mathrm{4,3}}&\:{K}_{\mathrm{4,4}}\end{array}\end{array}\right]$$

Here, odd-numbered rows (rows 1, 3)

$$\:{\mathrm{K}}_{\mathrm{1,1}}=\:EI{\left(\frac{\pi\:}{L}\right)}^{4}-\rho\:{v}^{2}{\left(\frac{\pi\:}{L}\right)}^{2}-m{\omega\:}_{n}^{2}$$

$$\:{\mathrm{K}}_{\mathrm{1,2}}=\frac{16\rho\:v{\omega\:}_{n}}{3L},\:{\mathrm{K}}_{\mathrm{1,4}}=\frac{32\rho\:v{\omega\:}_{n}}{15L}$$

$$\:{\mathrm{K}}_{\mathrm{3,3}}=\:EI{\left(\frac{3\pi\:}{L}\right)}^{4}-\rho\:{v}^{2}{\left(\frac{3\pi\:}{L}\right)}^{2}-m{\omega\:}_{n}^{2}$$

$$\:{\mathrm{K}}_{\mathrm{3,2}}=-\frac{48\rho\:v{\omega\:}_{n}}{5L},\:{\mathrm{K}}_{\mathrm{3,4}}=\frac{96\rho\:v{\omega\:}_{n}}{7L}$$.

Here, even rows (rows 2, 4)

$$\:{\mathrm{K}}_{\mathrm{2,2}}=\:EI{\left(\frac{2\pi\:}{L}\right)}^{4}-\rho\:{v}^{2}{\left(\frac{2\pi\:}{L}\right)}^{2}-m{\omega\:}_{n}^{2}$$

$$\:{\mathrm{K}}_{\mathrm{2,1}}=\frac{16\rho\:v{\omega\:}_{n}}{3L},\:{\mathrm{K}}_{\mathrm{2,3}}=-\frac{48\rho\:v{\omega\:}_{n}}{5L}$$

$$\:{\mathrm{K}}_{\mathrm{4,4}}=\:EI{\left(\frac{4\pi\:}{L}\right)}^{4}-\rho\:{v}^{2}{\left(\frac{4\pi\:}{L}\right)}^{2}-m{\omega\:}_{n}^{2}$$

$$\:{\mathrm{K}}_{\mathrm{4,1}}=\frac{32\rho\:v{\omega\:}_{n}}{15L}\:,\:{\mathrm{K}}_{\mathrm{4,3}}=\frac{96\rho\:v{\omega\:}_{n}}{7L}$$.

The mode shapes of the pipeline depend on the relative mode coefficients $\:{A}_{2j-1}$ and $\:{A}_{2k}$. After setting $\:{A}_{1}$ to 1, the other mode shape coefficients in Eqs. (33) and (35) are determined through iteration. Once coefficients $\:{A}_{2k}$ and $\:{A}_{2j-1}$ are established, Eqs. (33) and (35) become the mode frequency equations for system vibration. For example, due to the constraints of system damping, the pipe only exhibits the first two modes, i.e., for $\:j=1,{\mathrm{A}}_{2\mathrm{j}-1}={\mathrm{A}}_{1}$ (the amplitude of the first-order symmetric vibration mode), and for $\:{k=1,A}_{2k}={A}_{2}$ (the amplitude of the first-order asymmetric vibration mode). Therefore, Eqs. (33) and (35) simplify to Eqs. (42) and (43).

42 $$\:{A}_{1}\left[EI{\left(\frac{\pi\:}{L}\right)}^{4}-\rho\:{v}^{2}{\left(\frac{\pi\:}{L}\right)}^{2}-m{{\omega\:}_{1}}^{2}\right]=-\frac{2}{3}\left(\frac{8\rho\:v{\omega\:}_{1}}{L}\right){A}_{2}$$

43 $$\:{A}_{2}=-{A}_{1}\frac{16\rho\:v{\omega\:}_{1}}{3L\left[EI{\left(\frac{2\pi\:}{L}\right)}^{4}-\rho\:{v}^{2}{\left(\frac{2\pi\:}{L}\right)}^{2}-m{{\omega\:}_{1}}^{2}\right]}$$

Substituting $\:{\mathrm{A}}_{2}$ from formula (43) into formula (42) yields the frequency Formula (44).

44 $$\:\frac{3L\left[EI{\left(\frac{\pi\:}{L}\right)}^{4}-\rho\:{v}^{2}{\left(\frac{\pi\:}{L}\right)}^{2}-m{{\omega\:}_{1}}^{2}\right]}{16\rho\:v{\omega\:}_{1}}=\frac{16\rho\:v{\omega\:}_{1}}{3L\left[EI{\left(\frac{2\pi\:}{L}\right)}^{4}-\rho\:{v}^{2}{\left(\frac{2\pi\:}{L}\right)}^{2}-m{{\omega\:}_{1}}^{2}\right]}$$

Using Formula (44), we can determine the first natural frequency $\:{{\upomega\:}}_{1}$, as well as the mode amplitudes $\:{A}_{1}$ and $\:{A}_{2}$.

#### Engineering verification

**Fig. 5:**
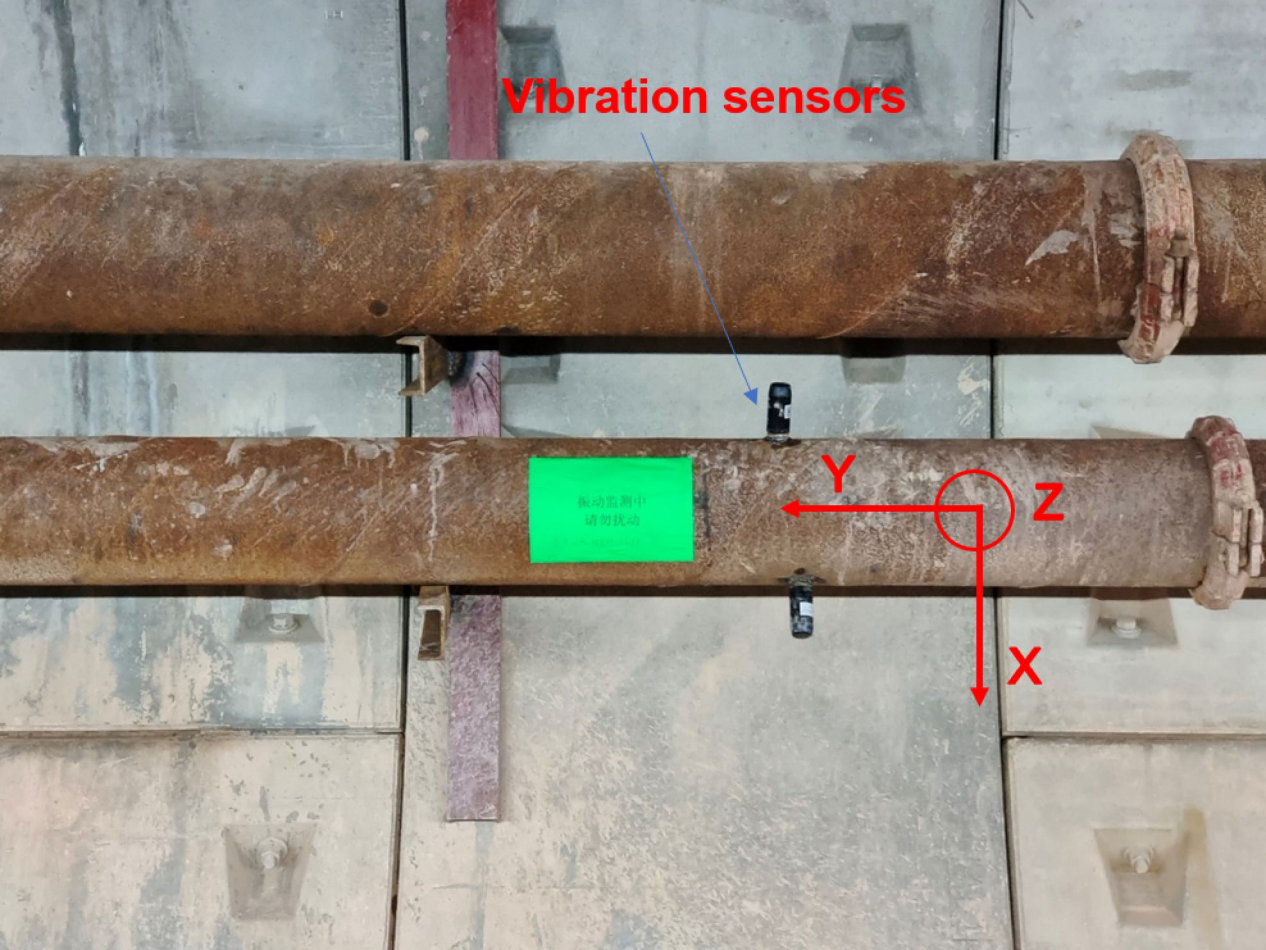
Photograph of the field vibration monitoring setup at the project site.

The water conveyance project from Tuancheng Lake to the Ninth Water Plant (Phase II) is a crucial component of Beijing’s South-to-North Water Diversion supporting project. The project starts at the water diversion point of the Tuancheng Lake regulation pool loop line and ends at the Longbeicun gate station of Phase I of the Tuanjiu project, with a total length of approximately 4 kilometers. It is the final section of the ‘one loop’ in Beijing’s South-to-North Water Diversion supporting project. The tunneling section involves complex geological conditions, sequentially passing through layers of gravel, hard rock, mixed soft and hard strata, and clay. Taking the measured data of point 8 in the X direction of the engineering project as an example (as shown in Fig. 5), compare the mode frequencies of pipeline vibration under different strata conditions with the calculated values.

Data Acquisition and Processing:

The field vibration monitoring system utilized iVS101 wireless vibration sensors to capture the vibration velocity signals of the pipeline at a sampling rate of 500 Hz. The signals were received by NY-R1 wireless data collectors and transmitted to the central data server via a Local Area Network (LAN) for storage and analysis. To identify the natural frequencies, the collected time-domain velocity signals were processed using the Fast Fourier Transform (FFT) to generate the frequency spectrum. The mode frequencies were then determined using the Peak Picking method based on the dominant peaks in the spectrum.

Protocol for Determination of Mass Parameters (*ρ* and *m*):

The fluid mass per unit length (*ρ*) is the critical model input. Since *ρ* represents a linear mass density (kg/m) rather than a volumetric density, it was derived based on a rigorous in-situ sampling protocol:

- Sampling Location: Slurry samples were collected from a sampling port 5 m downstream of the slurry pump.
- Timing and Frequency: Sampling was strictly conducted during the stable advancement phase. Three independent samples were collected at 10-minute intervals for each geological stratum.
- Sample Processing: To accurately capture the bulk properties of the two-phase flow, samples were not allowed to settle. They were subjected to mechanical agitation to ensure full homogenization. Large debris (particle diameter > 5 mm) was screened out to fit the measuring capacity of the equipment.
- Measurement and Calculation: The homogenized samples were tested using a standard mud balance to obtain the volumetric density (kg/m³). The model parameter *ρ* was then calculated by multiplying the arithmetic mean of these three.

density measurements by the pipeline’s inner cross-sectional area.

Calculation of Total Mass (*m*):

Consequently, the total mass per unit length (*m*) was obtained by summing the calculated fluid mass (*ρ*) and the constant pipe wall mass (derived from the steel density and the pipe’s geometric dimensions: outer diameter 270 mm, wall thickness 10 mm).

##### Modeling implication: equivalent continuum assumption

It is crucial to acknowledge that using a scalar fluid mass *ρ* mathematically transforms the discrete solid-liquid two-phase flow into an equivalent single-phase continuum. This modeling approach effectively captures the macroscopic inertial contribution of the slurry, which is the dominant factor governing the shift in the pipeline’s low-order natural frequencies. However, this homogenization implies a limitation: it inherently filters out the high-frequency discrete excitations and local wall pressure fluctuations caused by the random impact of large particles (in gravel strata) or mud clumps (in clay strata). While the current model accurately predicts the fundamental stability boundaries and global modal responses, capturing these transient, localized particle-impact dynamics would require the coupled CFD-DEM numerical methods discussed in the Introduction.

The geometric and physical parameters of the slurry straight pipe are shown in Table 1.

**Table 1:** Parameters of slurry discharge pipeline for tunnel boring machine.

Parameter category	Parameter name	Numerical	Unit
Geometric parameters	outer diameter $\:\left(D\right)$	270	mm
	inner diameter (*d*)	250	mm
	wall thickness	10	mm
	Span (support spacing) $\:\left(L\right)$	3600	mm
Material properties	Young’s modulus $\:\left(E\right)$	2.0 × 1011	Pa
	moment of inertia $\:\left(I\right)$	6.91 × 10 − 5	m⁴
	Bending stiffness $\:\left(EI\right)$	13.82 × 106	N·m²
Fluid parameter	Velocity $\:\left(v\right)$	3.5	m/s

1. Slurry circulation process

**Fig. 6:**
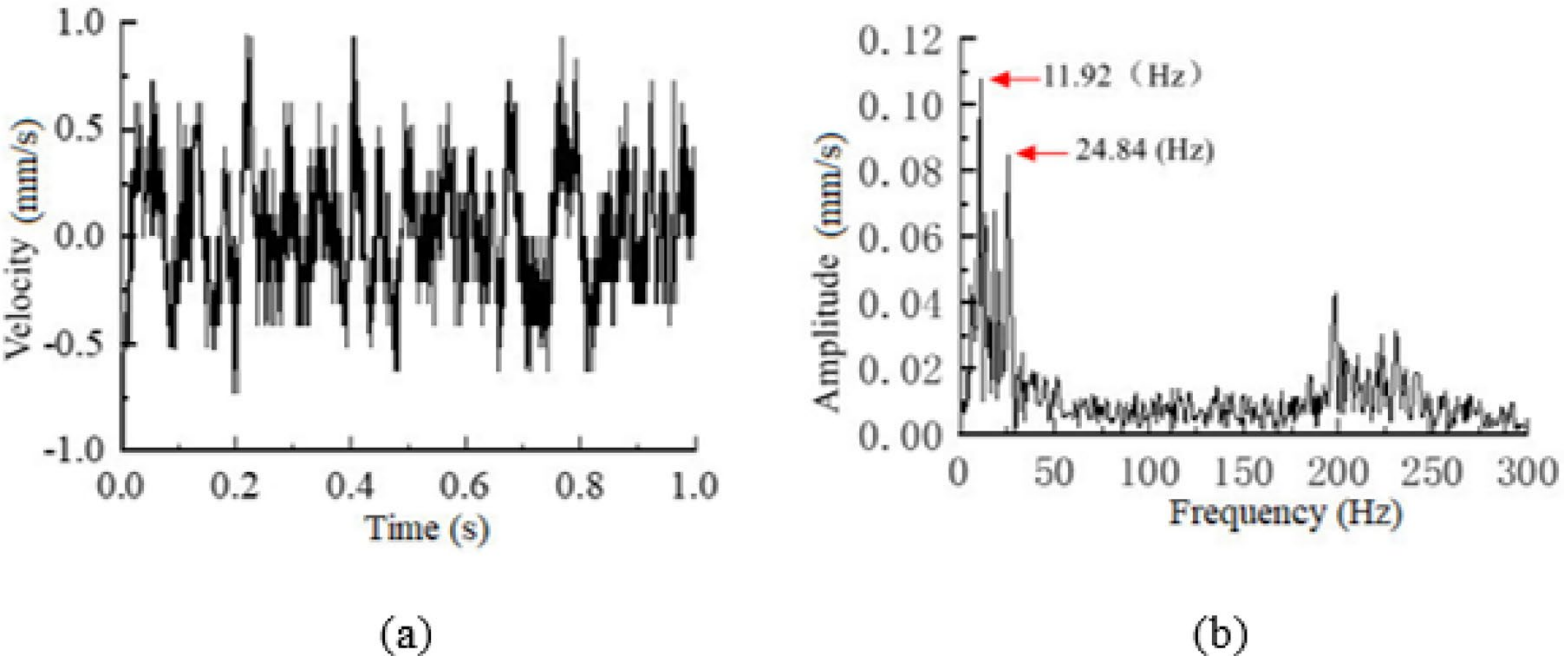
Vibration response in the X direction during the slurry circulation process. (**a**) Time-domain velocity signal: Real-time vibration velocity measured by iVS101 sensors; (**b**) FFT amplitude spectrum: Velocity amplitude spectrum obtained via Fast Fourier transform.

Before the shield machine begins advancement, the circulation system is activated in advance. At this stage, only slurry flows through the pipeline, with no large-sized rock debris transport. Under these conditions, the vibration characteristics of the pipeline under slurry turbulence excitation can be measured, and the vibration features of the pipeline solely induced by slurry turbulence can be analyzed. The measured data for vibration in the X direction are shown in Fig. 6. Based on the statistical analysis of the samples, the fluid mass per unit length *ρ* yielded a mean of 58.9 kg/m with a standard deviation (SD) of 0.85 kg/m (95% CI: [56.8, 61.0] kg/m). Consequently, the nominal total mass per unit length *m* is 123.04 kg/m. Propagating the 95% CI bounds of the fluid mass through the analytical model (Eq. 44) yields a predicted theoretical first-order natural frequency of 12.67 Hz, with an uncertainty bound (theoretical error bar) spanning from 12.57 Hz to 12.77 Hz. The measured fundamental frequency successfully falls within this theoretically propagated confidence band.

##### Determination of truncation order (*N*) and convergence study

In the theoretical calculations, the truncation order is set to *N* = 5. To rigorously justify the sufficiency of this truncation and demonstrate numerical stability, a modal convergence study was conducted by systematically increasing *N* from 3 to 10. Taking the slurry circulation process (Case 1) as a benchmark, the results reveal extremely rapid frequency convergence. For instance, the calculated fundamental frequency *f*_1_ shifts from 12.6710 Hz at *N* = 3, to 12.6702 Hz at *N* = 5, and remains stabilized at 12.6702 Hz at *N* = 10. Across the first five modes, the maximum relative frequency difference between the *N* = 5 and *N* = 10 truncation levels is strictly less than 0.002%.

Physically, this rapid convergence is governed by the structural characteristics of the Euler-Bernoulli beam, where the modal stiffness is proportional to the fourth power of the mode order (*n*^4^). The off-diagonal dynamic coupling (induced by the Coriolis force) between the lower-order modes and the modes beyond *N* = 5 is heavily suppressed by the immense impedance of the higher modes. Furthermore, as evidenced by the modal amplitudes in Table 3, the relative amplitude of the 5th-order mode (*A*_5_) has already decayed to the magnitude of 10^− 7^ compared to the fundamental mode (*A*_1_ = 1.0). Therefore, truncating the infinite-dimensional system at *N* = 5 successfully balances high computational precision with analytical efficiency, ensuring that the numerical solution is fundamentally converged.

##### Implications for simplified engineering design

From a practical engineering perspective, the extremely low magnitude of higher-order amplitudes (10^− 6^∼10^− 7^) suggests that the system’s dynamic behavior is overwhelmingly dominated by the fundamental symmetric and asymmetric modes. This implies that for routine design and stability checks, a simplified truncation to *N* = 2 (retaining only the first pair of modes) yields a frequency prediction that is sufficiently accurate for engineering purposes. Consequently, the analytical expression derived in Eq. (44), which is based on this 2-mode assumption, can serve as a reliable, rapid calculation tool for site engineers to estimate natural frequencies without resorting to complex matrix operations.

The first five mode natural frequencies and the relative mode amplitudes at the first-order mode frequency are shown in Tables 2 and 3.

**Table 2:** Natural frequencies of the first 5 mode orders (Calculated based on the coupled matrix model, Eq. 36).

Mode order	1	2	3	4	5
Natural frequency $\:f$ (Hz)	12.67	50.68	114.03	202.72	316.75

**Table 3:** Mode amplitudes A1 to A6($\:f=12.67\left(\mathrm{H}\mathrm{z}\right)$).

$\:{A}_{1}$	$\:{A}_{2}$	$\:{A}_{3}$	$\:{A}_{4}$	$\:{A}_{5}$	$\:{A}_{6}$
1.00000000	-0.00157815	-0.00000081	-0.00003713	-0.00000005	-0.00000470

The relative error between the measured and theoretical values of the first-order natural frequency is 6.29%. As the mode order increases, the natural frequency significantly rises from 12.67 Hz to 316.75 Hz, consistent with the Euler-Bernoulli beam theory’s characteristic of higher frequencies for higher-order modes. The amplitude of the first-order mode dominates, while the amplitudes of higher-order modes (2nd to 5th) rapidly decay, indicating that lower-order modes contribute most to system vibration. The amplitudes of higher-order modes approach zero, suggesting that under slurry turbulence excitation, the system primarily exhibits low-order vibrations.

(2) Clay stratum

**Fig. 7:**
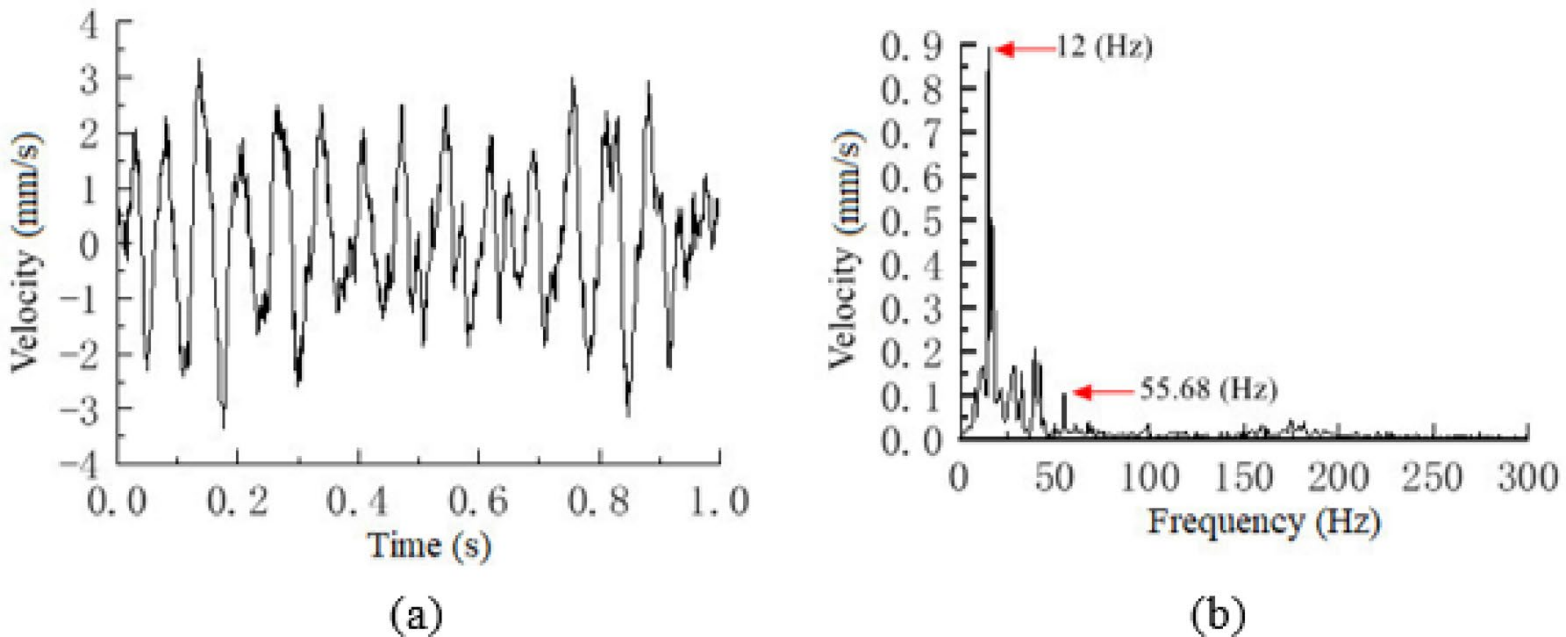
Vibration response in the X direction during the clay stratum. (**a**) Time-domain velocity signal: Real-time vibration velocity measured by iVS101 sensors; (**b**) FFT amplitude spectrum: Velocity amplitude spectrum obtained via Fast Fourier Transform.

During the advancement in cohesive soil strata, due to the adhesion and compression between the cutterhead and the soil, slurry cakes easily form at the cutterhead position. The block-like slurry cakes dislodged by the slurry move through the pipeline, forming large-diameter slurry balls. At this point, the pipeline vibrates under the combined stimulation of slurry turbulence and large-diameter slurry balls, with the X-direction vibration curve shown in Fig. 7. During the advancement in the clay stratum, the presence of slurry balls increased the measurement variance. The fluid mass per unit length *ρ* averaged 68.7 kg/m with an SD of 1.04 kg/m (95% CI: [66.1, 71.3] kg/m), resulting in a nominal total mass *m* of 132.84 kg/m. By propagating this density variance through the analytical model, the predicted first-order natural frequency is 12.19 Hz, with an associated theoretical uncertainty bound of [12.08 Hz, 12.30 Hz].

The first five mode natural frequencies and the relative mode amplitudes at the first mode frequency are shown in Tables 4 and 5.

**Table 4:** Natural frequencies of the first 5 mode orders (Calculated based on the coupled matrix model, Eq. 36).

Mode order	1	2	3	4	5
Natural frequency $\:f$ (Hz)	12.19	48.77	109.74	195.1	304.85

**Table 5:** Mode amplitudes A1 to A6($\:f=12.19\left(\mathrm{H}\mathrm{z}\right)$).

$\:{A}_{1}$	$\:{A}_{2}$	$\:{A}_{3}$	$\:{A}_{4}$	$\:{A}_{5}$	$\:{A}_{6}$
1.00000000	-0.00177101	-0.00000102	-0.00004167	-0.00000006	-0.00000527

The relative error between the measured and theoretical values of the first-order natural frequency is 1.58%. Compared to the slurry circulation process, the natural frequency slightly decreases (e.g., from 12.67 Hz to 12.19 Hz), which may be due to the slurry balls in the clay stratum increasing the effective mass of the system and reducing the pipe stiffness. The amplitude of the low-order mode (first order) still dominates, while the amplitude of the high-order modes remains very small, similar to the slurry circulation process. However, the second-order amplitude slightly increases (from − 0.00157815 to -0.00177101), suggesting that the slurry balls may have a slight enhancing effect on the mid-low order modes.

(3) Strata with soft upper layer and hard lower layer

**Fig. 8:**
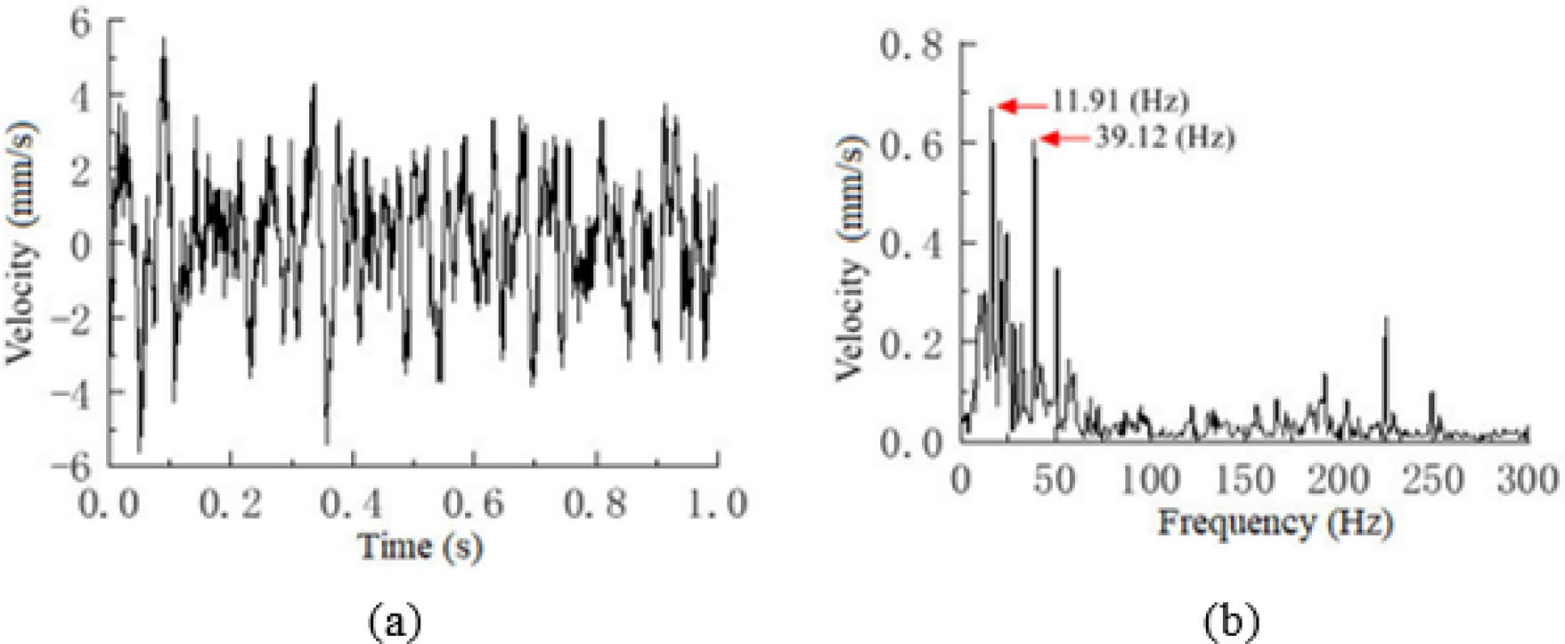
Vibration response in the X direction during strata with soft upper layer and hard lower layer. (**a**) Time-domain velocity signal: Real-time vibration velocity measured by iVS101 sensors; (**b**) FFT amplitude spectrum: Velocity amplitude spectrum obtained via Fast Fourier Transform.

During the advancement in strata with a soft upper layer and hard lower layer, the slurry transport pipeline carries a mixture of large-grained rock debris and slurry balls. Under the combined excitation of turbulent flow, large-grained rock debris, and slurry balls, the slurry transport pipeline vibrates. The X-direction vibration curve is shown in Fig. 8. In this complex mixed stratum, the highly heterogeneous mixture of gravel and slurry balls further increased the mass fluctuation. The sampled fluid mass *ρ* exhibited a mean of 78.5 kg/m with an SD of 1.65 kg/m (95% CI: [74.4, 82.6] kg/m), yielding a nominal total mass *m* of 142.64 kg/m. Propagating this wider 95% CI through the model provides a predicted first-order natural frequency of 11.76 Hz, with a corresponding theoretical error band of [11.59 Hz, 11.93 Hz].

The first five mode natural frequencies and the relative mode amplitudes at the first-order mode frequency are shown in Tables 6 and 7.

**Table 6:** The first five natural frequencies of mode orders (Calculated based on the coupled matrix model, Eq. 36).

Mode order	1	2	3	4	5
Natural frequency $\:f$ (Hz)	11.76	47.06	105.91	188.29	294.18

**Table 7:** Mode amplitudes A1 to A6($\:f=11.76\left(\mathrm{H}\mathrm{z}\right)$).

$\:{A}_{1}$	$\:{A}_{2}$	$\:{A}_{3}$	$\:{A}_{4}$	$\:{A}_{5}$	$\:{A}_{6}$
1.00000000	-0.00195229	-0.00000124	-0.00004593	-0.00000007	-0.00000581

The relative error between the measured and theoretical values of the first-order natural frequency is 1.26%. The further reduction in natural frequency (e.g., from 12.19 Hz to 11.76 Hz for the first order) may be due to the combined effect of large-grained debris and slurry balls, which increase mass and damping effects. The first mode remains dominant, but the amplitude of the second mode continues to increase (from − 0.00177101 to -0.00195229), indicating that the excitation by debris and slurry balls slightly enhances the vibration of mid-to-low-order modes, while the amplitudes of higher-order modes remain weak.

(4) Fully-sectioned hard rock stratum

**Fig. 9:**
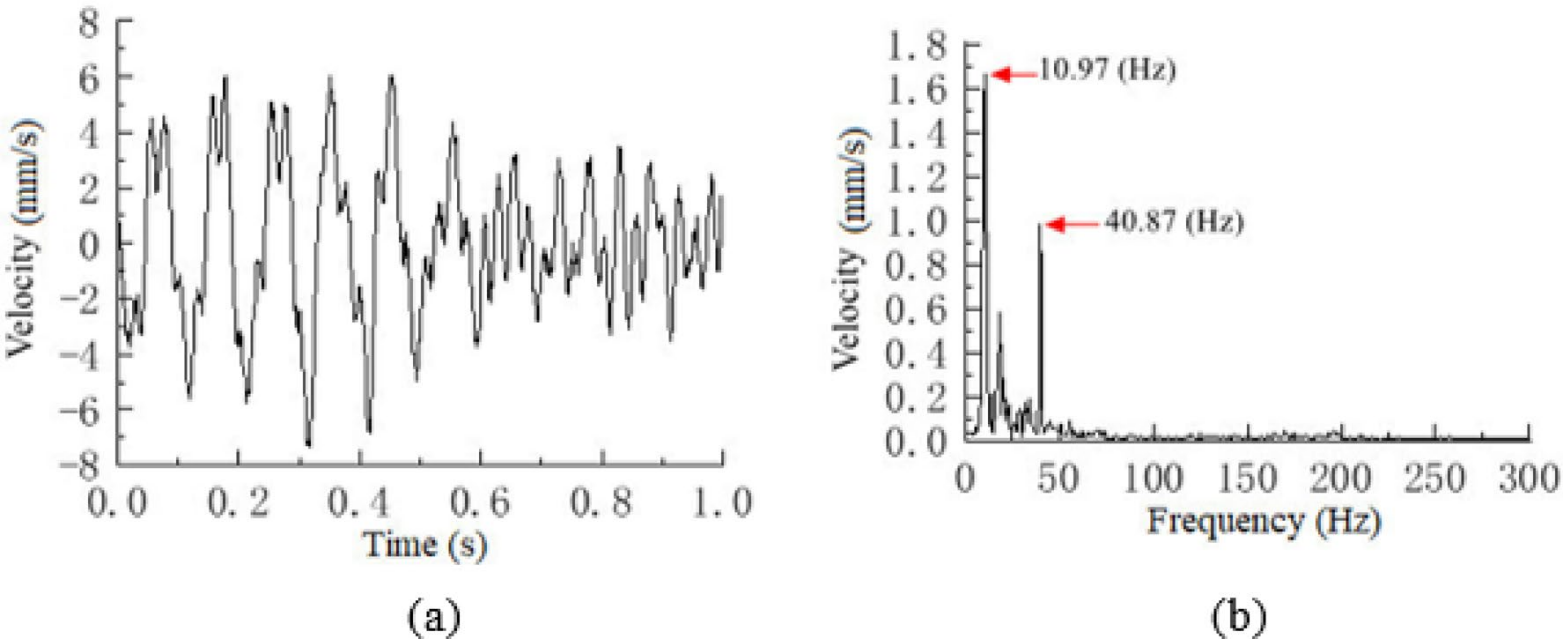
Vibration response in the X direction during fully-sectioned hard rock stratum. (**a**) Time-domain velocity signal: Real-time vibration velocity measured by iVS101 sensors; (**b**) FFT amplitude spectrum: Velocity amplitude spectrum obtained via Fast Fourier Transform.

During the advancement in fully intact hard rock strata, the transported medium within the pipeline consists of large-grained muck, with a very high content. Both the muck and turbulence serve as sources of excitation force, resulting in more intense pipeline vibrations. The actual measurement results for X-direction vibrations are shown in Fig. 9. During advancement in the fully intact hard rock stratum, the high content of large-grained rock debris led to the maximum mass variability. The fluid mass *ρ* yielded a mean of 88.4 kg/m with an SD of1.97 kg/m (95% CI: [83.5, 93.3] kg/m), bringing the nominal total mass *m* to152.54 kg/m. Incorporating these statistical bounds into the governing matrix equations produces a theoretical first-order natural frequency of 11.37 Hz, bounded by an uncertainty interval of [11.18 Hz, 11.56 Hz]. The consistent alignment between the measured field data and these theoretically propagated error bounds verifies the model’s predictive reliability under highly variable field conditions.

The first five mode natural frequencies and the relative mode amplitudes at the first mode frequency are shown in Tables 8 and 9.

**Table 8:** The first five natural frequencies of mode orders (Calculated based on the coupled matrix model, Eq. 36).

Mode order	1	2	3	4	5
Natural frequency $\:\mathrm{f}$ (Hz)	11.37	45.51	102.41	182.07	284.47

**Table 9:** Mode amplitudes A1 to A6($\:f=11.37\left(\mathrm{H}\mathrm{z}\right)$).

$\:{A}_{1}$	$\:{A}_{2}$	$\:{A}_{3}$	$\:{A}_{4}$	$\:{A}_{5}$	$\:{A}_{6}$
1.00000000	-0.00212562	-0.00000147	-0.00005001	-0.00000008	-0.00000633

The relative error between the measured and theoretical values of the first-order natural frequency is 3.65%. The natural frequency reaches its minimum value (e.g., the first order decreases to 11.37 Hz), which may be due to a high content of large-grain ballast in hard rock strata, further increasing the system’s mass and damping. The amplitude of the first mode remains dominant, while the amplitude of the second mode increases to -0.00212562, indicating that ballast excitation makes low-order mode vibrations more significant. The amplitudes of higher-order modes slightly increase (e.g., the fourth order rises from − 0.00004593 to -0.00005001), suggesting an increase in the complexity of system vibrations.

The observed relative errors (1.58%–6.29%) reflect the inherent complexities of the tunneling environment. Several factors contribute to these discrepancies: first, the assumption of perfectly rigid supports neglects the finite compliance of real-world steel brackets; second, the slurry is treated as a homogeneous continuum, which may not fully capture the transient non-homogeneity caused by localized debris clusters; and finally, environmental noise and high-frequency interference from the shield machine’s mechanical components may introduce minor deviations during field data acquisition.

#### Evaluation of noise robustness

In complex slurry shield tunneling environments, evaluating the noise robustness of the proposed method is essential. The robustness of this framework is theoretically and practically guaranteed across two dimensions:

1. Robustness against measurement noise

Field vibration signals are inevitably contaminated by high-frequency mechanical noise from TBM components. However, the data acquisition was executed at a constant sampling rate of 500 Hz, which yields a valid Nyquist bandwidth up to 250 Hz. This successfully captures the relevant low-order modes while preventing high-frequency aliasing. As proven by the theoretical model, the pipeline’s dynamic response is overwhelmingly dominated by the fundamental low-order modes (e.g., 11∼13 Hz), because higher-order modes possess exponentially higher stiffness (*n*^4^ scaling). This intrinsic structural property ensures a clear frequency separation between the pipeline’s fundamental resonance and high-frequency environmental noise. Consequently, the distinct spectral peaks of low-order modes in the FFT amplitude spectrum (Figs. 6, 7, 8 and 9) remain highly identifiable, making the frequency extraction process robust against severe background noise.

2. Robustness of the analytical model against input perturbations

Real-world operational “noise” also manifests as transient fluctuations in input parameters, such as instantaneous variations in slurry density due to localized gravel or clay clusters. Mathematically, the proposed coupled matrix equation (Eq. 36) and the derived analytical expression (Eq. 44) are structurally robust against such parameter noise. The natural frequency *ω*_*n*_ is a continuous algebraic function of the mass and centrifugal terms. Consequently, high-frequency random perturbations in the macroscopic fluid mass per unit length (*ρ*) or velocity (*v*) result only in bounded, continuous shifts in the predicted frequency rather than numerical divergence or computational instability. The equivalent continuum assumption inherently acts as a macroscopic low-pass filter, ensuring that the analytical model reliably predicts the global fundamental frequencies despite micro-scale slurry heterogeneity.

## Conclusions

This study successfully adapted and validated a classical fluid-structure interaction model for analyzing the lateral deflection and natural frequency of slurry shield muck discharge pipelines. By idealizing the pipeline as an infinite cylinder supported by equally spaced rigid rings and employing Euler-Bernoulli beam theory, the model effectively captures the dynamic interaction between the pipeline and flowing slurry. The primary contribution of this work lies not in theoretical derivation, but in the practical engineering application and rigorous field validation of key physical factors—such as vertical acceleration induced by slurry flow, Coriolis force, and curvature acceleration—providing deep insights into the vibration behavior of the pipeline under actual operating conditions across diverse geological strata.

1. Physical interpretation of FSI mechanisms:

Based on the derived Euler-Bernoulli beam model and Hamilton’s variational principle, the governing equation reveals two critical physical mechanisms driving the system’s behavior:

Negative stiffness effect (frequency reduction): The results consistently show that natural frequencies decrease as slurry flow velocity increases. Physically, this is governed by the centrifugal force term ($\:\rho\:{v}^{2}\frac{{\partial\:}^{2}\mathrm{z}}{\partial\:{x}^{2}}$). This term acts as an axial compressive load, introducing a negative stiffness effect that counteracts the pipeline’s structural bending stiffness (*EI*). As flow velocity rises, the effective stiffness of the system diminishes, leading to the observed drop in natural frequencies.

Validation against Classical Trends: This phenomenon aligns with the classical FSI trends reported in foundational literature, such as Ashley & Haviland (1950)^33^ and Païdoussis (1998)^27^ for pipes conveying fluid. Our results confirm that the slurry discharge pipeline exhibits the same monotonic frequency decay characteristic as standard water pipes. Furthermore, the magnitude of this reduction is physically plausible given the high density of the slurry (*ρ*_*slurry*_ > *ρ*_*water*_), which amplifies the centrifugal inertial effect (*ρv*^*2*^) compared to conventional water transport systems, thereby validating the predictive capability of the proposed model.

Modal stiffness scaling (low-order dominance): The engineering verification confirms that low-order modes dominate the vibration response. This is physically consistent with structural dynamics principles, where the modal stiffness of a beam is proportional to the fourth power of the mode order (*n*^4^). Consequently, higher-order modes possess exponentially higher stiffness, making them difficult to excite under the typical energy spectrum of slurry turbulence. From a practical engineering perspective, this suggests that vibration control and resonance avoidance strategies in tunneling projects can be effectively centered on the first few natural frequencies. This finding provides a practical insight: it validates the use of simplified, fast-solving analytical models for rapid design-stage scoping and real-time monitoring, significantly improving engineering efficiency over computationally expensive full-scale simulations.

(2) The variable separation method was employed to decompose the problem into spatial and temporal components, thereby determining the natural frequencies and mode shapes of the pipeline. Analysis indicates that the presence of slurry flow reduces the natural frequencies, and the system may exhibit instability at the critical flow velocity compared to the static state. Furthermore, the dynamic coupling between symmetric and asymmetric modes driven by slurry flow velocity and the Coriolis force was quantified using matrix methods, providing a systematic approach for calculating mode amplitudes and frequencies.
(3) The model’s validity was rigorously verified through comparisons with theoretical benchmarks and actual engineering data. In a case study of the water conveyance project from Tuancheng Lake to the Ninth Water Plant in Beijing, the relative error of the first-order mode natural frequency ranged from 1.58% to 6.29%. The model was applied under different geological conditions, such as gravel, hard rock, transitions between hard and soft strata, and clay layers. The results showed that an increase in fluid mass per unit length (due to slurry clumps or large debris) led to a reduction of approximately 3% to 5.5% in the natural frequency, and a slight increase in the amplitude of the second mode. The amplitude of the high-order mode is extremely small, confirming that the low-order mode dominates in the vibration response.
(4) The findings of this study have significant implications for the design and operation of slurry shield muck discharge pipelines. By accurately predicting natural frequencies and identifying dominant vibration modes, engineers can take targeted measures, such as optimizing support spacing or selecting appropriate materials, to mitigate excessive vibrations. The model is adaptable to different geological conditions and slurry compositions, further enhancing its practicality in diverse tunnel environments. In summary, this research provides a valuable tool for ensuring the structural integrity and operational efficiency of pipeline systems in tunnel construction projects.

(5) Limitations and scope of applicability:

It is essential to frankly discuss the impact of the model’s geometric idealizations:

Rigid support assumption: The model assumes the pipeline is supported by ‘rigid rings.’ In actual engineering, the steel brackets and tunnel segments possess finite stiffness. Introducing real-world support flexibility would decrease the effective stiffness of the system, theoretically leading to lower natural frequencies than those predicted. Therefore, the current model provides a theoretical upper-bound estimate for the structural stiffness. It should be noted that while the model generally over-predicts the frequency (e.g., Cases 1, 2, and 4), a slight under-prediction was observed in Case 3 (1.26% error). This suggests that in complex strata (e.g., soft upper and hard lower layers), the dynamic coupling between the high-concentration rock debris and the pipe wall may introduce a ‘pseudo-stiffness’ effect or fluctuations in instantaneous mass density that can cause the measured frequency to deviate slightly from the theoretical upper bound. Overall, the relative error of 1.58% to 6.29% confirms that the model serves as a reliable and conservative baseline for engineering design.

Pipeline junctions: The ‘infinitely long cylinder’ assumption simplifies the periodic structure of the pipeline but neglects the localized effects of flange joints. In practice, these junctions may introduce rotational flexibility and additional damping, potentially causing slight deviations in the mode shapes (particularly nodal positions) compared to the ideal continuous beam. Future research should consider finite element modeling of these joints to refine the mode shape predictions.

## Data Availability

The data that support the findings of this study are available from the corresponding author upon reasonable request.

## Appendix A: Derivation of fourier coefficients

The odd extension of the function $\:\mathrm{cos}\left(\left(2j-1\right)\frac{{\uppi\:}x}{L}\right)$ results in formula (A1).

A1 $$\:cos\left(\left(2j-1\right)\frac{\pi\:x}{L}\right)=\left\{\begin{array}{c}{cos}\left(\left(2j-1\right)\frac{\pi\:x}{L}\right),0\le\:x\le\:L\\\:-{cos}\left(\left(2j-1\right)\frac{\pi\:x}{L}\right),-L\le\:x<0\end{array}\right.$$

For the odd extension of $\:cos\left(\left(2j-1\right)\frac{\pi\:x}{L}\right)$, approximate it using a sine series and determine the coefficients $\:{a}_{k}$ to satisfy the formula shown in (A2).

A2 $$\:cos\left(\left(2j-1\right)\frac{\pi\:x}{L}\right)=\sum\:_{k=1}^{\infty\:}{a}_{k}{sin}\left(k\frac{\pi\:x}{L}\right)$$

Here, $\:{a}_{k}$ represents the coefficients of the sine series terms, calculated using the formula shown in Formula (A3).

A3 $$\:{a}_{k}=\frac{1}{L}{\int\:}_{-L}^{L}{cos}\left(\left(2j-1\right)\left(\frac{\pi\:}{L}\right)x\right){sin}\left(k\frac{\pi\:}{L}x\right)dx$$

For convenience of calculation, let

A4 $$\:{a}_{k}=S\left(x\right)$$

The integration interval $\:\left[-L,L\right]$ is divided into segments as follows

A5 $$\:S\left(x\right)=\frac{1}{L}{\int\:}_{0}^{L}{cos}\left(\left(2j-1\right)\left(\frac{\pi\:}{L}\right)x\right){sin}\left(k\frac{\pi\:}{L}x\right)dx-\frac{1}{L}{\int\:}_{-L}^{0}{cos}\left(\left(2j-1\right)\left(\frac{\pi\:}{L}\right)x\right){sin}\left(k\frac{\pi\:}{L}x\right)dx$$

Using trigonometric identities, we can derive formula (A6).

A6 $$\:cosAsinB=\frac{1}{2}\left[{sin}\left(A+B\right)-{sin}\left(A-B\right)\right]$$

By combining Eqs. (A5) and (A6), we can obtain Formula (A7).

A7 $$\:{cos}\left(\left(2j-1\right)\left(\frac{\pi\:}{L}\right)x\right){sin}\left(k\frac{\pi\:}{L}x\right)=\frac{1}{2}[{sin}\left(\left(2j-1+k\right)\left(\frac{\pi\:}{L}\right)x\right)-sin(\left(2j-1-k\right)\left(\frac{\pi\:}{L}\right)x\left)\right]$$

Substituting the relationship (A7) into formula (A5) and performing the integration over the interval $\:\left[0,L\right]$ first, we obtain formula (A8).

A8 $$\:{S}_{\left(0,L\right)}=\frac{1}{2L}{\int\:}_{0}^{L}[sin(\left(2j-1+k\right)\left(\frac{\pi\:}{L}\right)x)-sin(\left(2j-1-k\right)\left(\frac{\pi\:}{L}\right)x\left)\right]dx$$

This integral can be broken down into two parts, each of which can be calculated directly. For the function $\:\mathrm{s}\mathrm{i}\mathrm{n}\left(ax\right)$, its indefinite integral is$\:-\frac{1}{a}\mathrm{cos}\left(ax\right)$, where $\:a\ne\:0$. Therefore, for each sine function, the formula is shown in (A9).

A9 $$\:{\int\:}_{0}^{L}{sin}\left(ax\right)dx=-\frac{1}{a}cos\left(ax\right)\left|\begin{array}{c}L\\\:0\end{array}=-\frac{1}{a}\right.[{cos}\left(aL\right)-cos(0\left)\right]$$

Using formula (A9) to calculate the integral expression (A8), and noting that $\:\mathrm{cos}\left(0\right)=1$, we obtain formula (A10).

A10 $$\:{S}_{\left(0,L\right)}=\frac{1}{2L}\left\{-\frac{1}{\left(2j-1+k\right)\frac{\pi\:}{L}}\left[{cos}\left(\left(2j-1+k\right)\left(\pi\:\right)\right)-1\right]+\frac{1}{\left(2j-1-k\right)\left(\frac{\pi\:}{L}\right)}[{cos}\left(\left(2j-1-k\right)\left(\pi\:\right)\right)-1]\right\}$$

Further simplification of formula (A10) yields formula (A11).

A11 $$\:{S}_{\left(0,L\right)}=\frac{1}{2\pi\:}\left\{-\frac{1}{\left(2j-1+k\right)}\left[{cos}\left(\left(2j-1+k\right)\left(\pi\:\right)\right)-1\right]+\frac{1}{\left(2j-1-k\right)}[{cos}\left(\left(2j-1-k\right)\left(\pi\:\right)\right)-1]\right\}$$

Since both *j* and *k* are positive integers, 2*j* is twice *j*, so 2*j* must be even. Subtracting 1 from an even number results in an odd number, meaning 2*j*-1 is odd. Subtracting or adding *k* to this odd number depends on whether *k* is odd or even. Therefore, we get expression (A12).

A12 $$\:\left\{\begin{array}{c}{cos}\left(\left(2j-1+k\right)\left(\pi\:\right)\right)=-1,k\:is\:an\:even\:EquationNumber\\\:{cos}\left(\left(2j-1+k\right)\left(\pi\:\right)\right)=1,\:k\:is\:an\:odd\:EquationNumber\\\:\begin{array}{c}{cos}\left(\left(2j-1-k\right)\left(\pi\:\right)\right)=-1,k\:is\:an\:even\:EquationNumber\\\:{cos}\left(\left(2j-1-k\right)\left(\pi\:\right)\right)=1,k\:is\:an\:odd\:EquationNumber\end{array}\end{array}\right.$$

Using Formula (A12), Formula (A11) is further simplified to Eqs (A13a) and (A13b).

When k is even,

A13a $$\:S_{{\left( {0,L} \right)}} = \frac{1}{{2\pi \:}}\left[ { - \frac{1}{{\left( {2j - 1 + k} \right)}}( - 1 - 1) + \frac{1}{{\left( {2j - 1 - k} \right)}}( - 1 - 1)} \right]$$

When k is an odd number,

A13b $$\:S_{{\left( {0,L} \right)}} = \frac{1}{{2\pi \:}}\left[ { - \frac{1}{{\left( {2j - 1 + k} \right)}}(1 - 1) + \frac{1}{{\left( {2j - 1 - k} \right)}}(1 - 1)} \right]$$

Using formulas (A13a) and (A13b), we can derive formula (A14).

A14 $$\:S_{{\left( {0,L} \right)}} = \left\{ {\begin{array}{*{20}c} {\frac{1}{{\pi \:}}\frac{{2k}}{{\left( k \right)^{2} - \left( {2j - 1} \right)^{2} }},k\:is\:an\:even\:Equation\;Number} \\ {\:0,k\:is\:an\:odd\:Equation\;Number} \\ \end{array} } \right.$$

For the integral over the interval $\:\left[-L,0\right]$, the integral expression (A15) is obtained.

A15 $$\:{S}_{\left(-L,0\right)}=\frac{1}{L}{\int\:}_{-L}^{0}{cos}\left(\left(2j-1\right)\left(\frac{\pi\:}{L}\right)x\right){sin}\left(k\left(\frac{\pi\:}{L}\right)x\right)dx$$

Following the processing steps of formulas (A6) to (A13a) and (A13b), we can obtain the simplified result of this integral expression, as shown in formula (A16).

A16 $$\:S_{{\left( { - L,0} \right)}} = \left\{ {\begin{array}{*{20}c} { - \frac{1}{{\pi \:}}\frac{{2k}}{{\left( k \right)^{2} - \left( {2j - 1} \right)^{2} }},k\:is\:an\:even\:EquationNumber} \\ {\:0,k\:is\:an\:odd\:Equation\;Number} \\ \end{array} } \right.$$

The complete integral expression for $\:\mathrm{S}\left(x\right)$ results in Formula (A17).

A17 $$\:S\left(x\right)={S}_{\left(0,L\right)}-{S}_{\left(-L,0\right)}$$

After rearranging, we obtain Formula (A18).

A18 $$\:S\left( x \right) = \left\{ {\begin{array}{*{20}c} {\frac{1}{{\pi \:}}\frac{{2k}}{{\left( k \right)^{2} - \left( {2j - 1} \right)^{2} }} - \left[ { - \frac{1}{{\pi \:}}\frac{{2k}}{{\left( k \right)^{2} - \left( {2j - 1} \right)^{2} }}} \right],k\:is\:an\:even\:Equation\;Number} \\ {\:0,k\:is\:an\:odd\:EquationNumber} \\ \end{array} } \right.$$

After organizing, we obtain formula (A19).

A19 $$\:{a}_{k}=\left\{\begin{array}{c}\frac{4}{\pi\:}\frac{K}{{\left(k\right)}^{2}-{\left(2j-1\right)}^{2}},k\:is\:an\:even\:EquationNumber\\\:0,k\:is\:an\:odd\:EquationNumber\end{array}\right.$$

For the odd extension of the function $\:\mathrm{c}\mathrm{o}\mathrm{s}\left(2k\frac{\pi\:x}{L}\right)$, we need to solve for the coefficients $\:{a}_{j}$ to satisfy Formula (A20).

A20 $$\:cos\left(2k\frac{\pi\:x}{L}\right)=\sum\:_{j=1}^{\infty\:}{a}_{j}sin\left(j\frac{\pi\:x}{L}\right)$$

Here, $\:{a}_{j}$ represents the coefficients of the sine series terms, calculated according to formula (A21).

A21 $$\:{a}_{j}=\frac{1}{L}{\int\:}_{-L}^{L}{cos}\left(2k\left(\frac{\pi\:}{L}\right)x\right){sin}\left(j\left(\frac{\pi\:}{L}\right)x\right)dx$$

From the properties of odd extension, we can derive formula (A22).

A22 $$\:{cos}\left(2k\frac{\pi\:x}{L}\right)=\left\{\begin{array}{c}{cos}\left(2k\frac{\pi\:x}{L}\right),0\le\:x\le\:L\\\:-{cos}\left(2k\frac{\pi\:x}{L}\right),-L\le\:x<0\end{array}\right.$$

Using formula (A22), the integral expression (A21) is integrated in segments to obtain formula (A23).

A23 $$\:{a}_{j}=\frac{1}{L}\left[{\int\:}_{0}^{L}{cos}\left(2k\left(\frac{\pi\:}{L}\right)x\right){sin}\left(j\left(\frac{\pi\:}{L}\right)x\right)dx-{\int\:}_{-L}^{0}{cos}\left(2k\left(\frac{\pi\:}{L}\right)x\right){sin}\left(j\left(\frac{\pi\:}{L}\right)x\right)dx\right]$$

After rearranging formula (A23), we obtain formula (A24).

$$\:{a}_{j}=\frac{1}{2L}\left[-\frac{1}{\left(2k+j\right)\frac{\pi\:}{L}}{cos}\left(\left(2k+j\right)\frac{\pi\:x}{L}\right)\left|\begin{array}{c}L\\\:0\end{array}+\frac{1}{\left(2k-j\right)\frac{\pi\:}{L}}{cos}\left(\left(2k-j\right)\frac{\pi\:x}{L}\right)\left|\begin{array}{c}L\\\:0\end{array}\right.\right.\right]$$

A24 $$\:-\frac{1}{2L}\left[-\frac{1}{\left(2k+j\right)\frac{\pi\:}{L}}{cos}\left(\left(2k+j\right)\frac{\pi\:x}{L}\right)\left|\begin{array}{c}0\\\:-L\end{array}+\frac{1}{\left(2k-j\right)\frac{\pi\:}{L}}{cos}\left(\left(2k-j\right)\frac{\pi\:x}{L}\right)\left|\begin{array}{c}0\\\:-L\end{array}\right.\right.\right]$$

Since both *j* and *k* are positive integers, 2*k* is twice *k*, so 2*k* must be even. When *j* is even, (2*k* ± *j*) is even; when *j* is odd, (2*k* ± *j*) is odd. Therefore, we can derive formula (A25).

A25 $$\:\left\{\begin{array}{c}{cos}\left(\left(2k+j\right)\left(\pm\:\pi\:\right)\right)=1,\:j\:is\:an\:even\:EquationNumber\\\:{cos}\left(\left(2k-j\right)\left(\pm\:\pi\:\right)\right)=1,j\:is\:an\:even\:EquationNumber\\\:\begin{array}{c}{\:cos}\left(\left(2k+j\right)\left(\pm\:\pi\:\right)\right)=-1,j\:is\:an\:odd\:EquationNumber\\\:{\:cos}\left(\left(2k-j\right)\left(\pm\:\pi\:\right)\right)=-1,j\:is\:an\:odd\:EquationNumber\end{array}\end{array}\right.$$

Using the relational expression (A25), we can further simplify formula (A24) to obtain formulas (A26a) and (A26b).

When *j* is an even number,

A26a $$\begin{aligned} \:a_{j} & = \frac{1}{{2\pi \:}}\left[ { - \frac{1}{{\left( {2k + j} \right)}}\left( {1 - 1} \right) + \frac{1}{{\left( {2k - j} \right)}}\left( {1 - 1} \right)} \right] \\ & - \frac{1}{{2\pi \:}}\left[ { - \frac{1}{{\left( {2k + j} \right)}}\left( {1 - 1} \right) + \frac{1}{{\left( {2k - j} \right)}}\left( {1 - 1} \right)} \right] \\ \end{aligned}$$

When *j* is an odd number,

A26b $$\begin{aligned} \:a_{j} & = \frac{1}{{2\pi \:}}\left[ { - \frac{1}{{\left( {2k + j} \right)}}\left( { - 1 - 1} \right) + \frac{1}{{\left( {2k - j} \right)}}\left( { - 1 - 1} \right)} \right] \\ & - \frac{1}{{2\pi \:}}\left[ { - \frac{1}{{\left( {2k + j} \right)}}\left( {1 - ( - 1)} \right) + \frac{1}{{\left( {2k - j} \right)}}\left( {1 - ( - 1)} \right)} \right] \\ \end{aligned}$$

After rearranging formulas (A26a) and (A26b), we obtain formula (A27).

A27 $$\:{a}_{j}=\left\{\begin{array}{c}\frac{4}{\pi\:}\frac{j}{{\left(j\right)}^{2}-{\left(2k\right)}^{2}},j\:is\:an\:odd\:EquationNumber\\\:0,j\:is\:an\:even\:EquationNumber\end{array}\right.$$

## List of symbols

*D*: Outer diameter of the slurry discharge pipeline (m)
*d*: Inner diameter of the pipeline (m)
*E*: Young’s modulus of the pipeline material (Pa)
*I*: Moment of inertia of the pipe cross-section (m^4^)
*L*: Span length (distance between supports) (m)
*m*: Total mass per unit length (pipeline + slurry) (kg/m)
*t*: Time variable (s)
*v*: Axial flow velocity of the slurry (assumed constant) (m/s)
*x*: Axial spatial coordinate along the pipeline (m)
*z*(*x*,*t*): Lateral deflection (displacement) of the pipeline (m)
*A*_*n*_: Mode amplitude coefficient of the *n*-th mode
*K*(*ω*_*n*_): Coefficient matrix for the coupled vibration equations
*N*: Truncation order for the mode superposition method
*ρ*: Fluid (slurry) mass per unit length (kg/m)
*ω*_*n*_: Natural angular frequency of the *n*-th mode (rad/s)
*f*: Natural frequency (H_z_)
*λ*: Eigenvalue parameter in the separation of variables
